# Unexpected migration patterns in a high-latitude breeding songbird: evidence from multi-sensor geolocators and isotopes

**DOI:** 10.1186/s40462-025-00618-6

**Published:** 2025-12-20

**Authors:** Stephanie J. Szarmach, Johanna K. Beam, Mads Moore, Benjamin M. Van Doren, Alan Brelsford, David P. L. Toews

**Affiliations:** 1 Department of Biology, Pennsylvania State University, University Park, Pennsylvania, USA; 2 Migratory Bird Center, Smithsonian’s National Zoo and Conservation Biology Institute, Washington, DC USA; 3 Department of Ecology, Evolution, and Environmental Biology, Columbia University, New York, NY USA; 4 Department of Natural Resources and Environmental Sciences, University of Illinois Urbana-Champaign, Urbana, IL USA; 5 Department of Evolution, Ecology, and Organismal Biology, University of California Riverside, Riverside, CA USA

**Keywords:** Seasonal migration, Avian migratory behavior, Barometric geolocators, Yellow-rumped warbler, Flight altitude, Evolution of migration routes

## Abstract

**Background:**

Migratory birds often exhibit within-species variation in migration routes and non-breeding areas, yet the mechanisms shaping these patterns remain poorly understood, particularly in high-latitude breeding populations. Several hypotheses have been proposed to explain why birds follow particular routes: optimal migration theory proposes that routes minimizing time or energy expenditure are favored, whereas the historical contingency hypothesis posits that routes are shaped by past range expansion, sometimes resulting in “suboptimal” migrations. We investigated whether distance minimization or historical contingency more strongly influenced migration routes in high-latitude breeding myrtle warblers (*Setophaga coronata coronata*), which indirect evidence previously suggested follow a shorter route to the Pacific Coast rather than the core Gulf Coast nonbreeding area.

**Methods:**

We tracked the migrations of six Alaskan myrtle warblers using geolocators measuring both light and atmospheric pressure and inferred nonbreeding areas using hydrogen isotopes for a larger sample of birds breeding in Alaska, British Columbia, and Alberta (*n* = 167). Additionally, we compared migration tracks derived from light-level data exclusively with those that incorporated atmospheric pressure.

**Results:**

Contrary to expectations, all geolocator-tracked birds and most with stable isotope data migrated to the southeastern United States, with just 5% of individuals possibly wintering on the Pacific Coast. Using pressure data allowed us to resolve migration routes and timing more precisely than traditional light-level methods, while also elucidating flight altitude and fine-scale elevational movements.

**Conclusions:**

We found that myrtle warblers breeding in northwestern North America migrate farther than previously thought, despite being generally regarded as a relatively short-distance migrant. Our findings contradict previous studies that suggested myrtle warblers breeding in Alaska and northern British Columbia typically follow a shorter migration route to the Pacific Coast. This seemingly suboptimal route—similar to routes followed by the few other songbirds tracked from the region—is consistent with the historical contingency hypothesis, which proposes that migration routes reflect past range expansions. We recommend that researchers conducting geolocation studies leverage tags with barometers, as the additional atmospheric pressure data greatly improved our ability to characterize migration at a fine scale over the full annual cycle.

**Supplementary Information:**

The online version contains supplementary material available at 10.1186/s40462-025-00618-6.

## Background

Migratory animals travel between distinct breeding and nonbreeding ranges seasonally to exploit spatial and temporal heterogeneity in resource availability, predation, and competition while escaping unfavorable environmental conditions [1, 2]. In birds, recaptures of marked individuals, genetic data, and, increasingly, remote tracking have revealed intraspecific variation in migration routes and nonbreeding locations [3–5]. Several hypotheses have been proposed to explain observed variation in migration routes. “Optimal migration theory” postulates that migration strategies evolved to minimize either migration time or energy expenditure [6, 7]. Although the energetic cost of a migration route depends on many factors, such as weather, topography, stopover habitat, and predation [7], shorter, more direct, migration routes are typically expected to reduce migration time and energy expenditure compared to substantially longer routes. In line with this expectation, in many species nonbreeding longitude is correlated with breeding longitude (i.e., birds breeding in the west winter farther west; [8–10]) and models that minimize migration distance as a proxy for energy expenditure accurately predict empirical patterns of migratory connectivity [9]. However, in some cases birds follow nondirect seemingly “sub-optimal’ migration routes, traveling across a continent—or even between continents—rather than to a longitudinally closer nonbreeding area [3, 10, 11]. Such sub-optimal migration routes have been explained by the “historical contingency” hypothesis, which posits that migration routes retrace paths of historical range expansion following the retreat of glaciers after the Last Glacial Maximum (LGM [3, 12]). This hypothesis was first proposed to explain why coastal and inland populations of the Swainson’s thrush (*Catharus ustulatus*) follow dramatically different migration routes and form a migratory divide in British Columbia, where individuals within the hybrid zone migrate different directions [3, 13].

These two proposed mechanisms differ in the degree of flexibility or constraint expected to shape avian migration routes, which has implications for how migration routes evolve as a species’ range shifts. The historical contingency hypothesis implies that migration routes are constrained to follow the paths of past range expansion—likely due to a combination of genetic and environmental factors—and suggests that the evolution of new migration routes is rare, even when the historical route is suboptimal [10, 14]. Alternatively, optimal migration theory predicts that selective pressures to minimize energy expenditure or migration time would promote the establishment of more efficient routes as a range shifts [9]. Although, in reality, both historical processes and energetic optimization likely influence migration routes in combination, unraveling the relative contribution of each mechanism elucidates how evolutionary contingency and selection shape complex behavioral traits and influence whether migration routes shift with changes in geographic range.

Conversely, migration strategies may directly influence the spatial distribution of species, as the degree of flexibility or constraint shaping migration routes could either facilitate or restrict range expansion. Studies have found evidence that long-distance migration constrains range expansion in some taxa, both historically [15–18] and within the last half century [19]. For some North American migratory birds, simulated breeding ranges based on resource availability extend much farther north and west than species’ true ranges [18, 20]. These findings imply that range expansion into northwestern North America may be constrained in migratory species whose routes are largely ruled by historical contingency, because breeding farther from the nonbreeding ground could lead to longer migration routes that are too energetically costly. However, other species show no association between migratory distance and range size [21], and do breed in high latitude regions far from core nonbreeding areas. This could be facilitated by the evolution of more efficient migration routes to closer nonbreeding areas as a species’ range expands. Migration routes to new nonbreeding areas (and, conversely, to new breeding grounds from an existing nonbreeding area [22]) have evolved in a number of avian species, even within the last fifty years [23, 24].

Linking the breeding and nonbreeding grounds of individual birds is essential for characterizing intraspecific variation in migratory behavior and inferring the processes shaping migration routes. Investigating the migrations of birds breeding at high-latitude range edges is especially valuable for understanding the interplay between migration routes and range expansion, yet few studies have tracked songbirds breeding at high latitudes in North America (but see [25, 26]). Here, we test whether distance minimization (as one proxy of energy optimization) or historical contingency plays a stronger role in shaping the migration routes of a high-latitude breeding songbird, the myrtle warbler (*Setophaga coronata coronata*; a subspecies within the yellow-rumped warbler species complex). Myrtle warblers belong to the parulid warbler family—a valuable system for studying the relationship between post-glacial range expansion into the high latitudes of western North America and variation in migratory strategies. During the LGM, many boreal-breeding birds—including warblers—are thought to have occupied a shared refugium in the southeastern United States, with their ranges expanding northward and westward after the retreat of glaciers [12, 27, 28]. In the present day, many warblers breeding in the North American boreal forest occupy ranges that are narrower than the extent of available habitat [18], and species that migrate farther tend to have smaller ranges [21]. Although this suggests that migration distance constrains range expansion in this group, a few species do range broadly from the Atlantic Coast to Alaska, including the myrtle warbler [18].

**Fig. 1:**
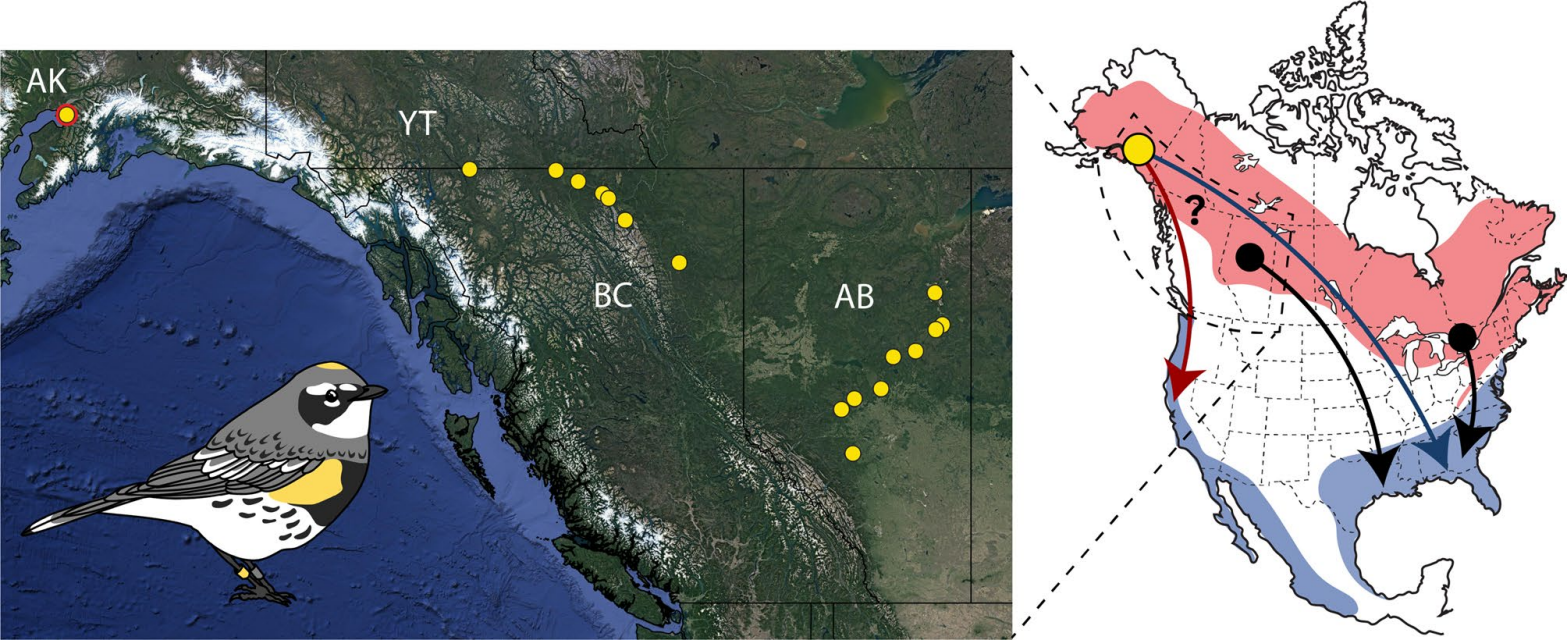
(Left) Sampling map depicting where geolocators were deployed (Anchorage, AK; the point with red outline) and where feather samples were collected for stable isotope analysis (yellow points). Base map: Google Satellite Hybrid (obtained through QuickMapServices QGIS plugin), retrieved 25 January 2024. (Right) Schematic representing predicted migration routes and nonbreeding areas for high-latitude breeding myrtle warblers (*Setophaga coronata coronata*) according to two alternate hypotheses. The myrtle warbler breeding range is shown in red and nonbreeding range in blue. Known connectivity between breeding and nonbreeding areas based on band recoveries are represented by black arrows. The yellow dot indicates where geolocators were deployed in the present study (Anchorage, AK). The red arrow depicts a predicted migration route based on the distance minimization hypothesis, in which warblers follow a shorter, more direct route to the closest nonbreeding area. The blue area shows a predicted migration route based on the historical contingency hypothesis, in which the migration route retraces the path of past range expansion to the core Gulf Coast nonbreeding area

The myrtle warbler has two disjunct nonbreeding ranges—one on the Gulf Coast extending to the Atlantic Coast and the other along the Pacific Coast (Fig. 1). The myrtle warblers that winter on the Pacific Coast were first described as a subspecies, *S. c. hooveri*, based on their longer wings and tails compared to eastern myrtle warblers, and in this original description it was predicted that they bred in Alaska and British Columbia [29, 30]. Although *S. c. hooveri* is not typically regarded as a subspecies today, analysis by Toews et al. [31] of morphological variation and stable isotopes in myrtle warblers migrating through southwestern British Columbia supported the original prediction that the birds wintering on the Pacific Coast breed at high latitudes in northwestern North America. This suggests that myrtle warblers, commonly considered short-distance migrants (e.g [32, 33]), evolved a shorter, more direct migration route to a closer nonbreeding area as their breeding range expanded into the high latitudes of western North America. However, because Toews et al. [34] sampled myrtle warblers during migration, rather than from the breeding range, further study is needed to determine whether all myrtle warblers breeding at high western latitudes migrate to the Pacific Coast. Sampling from the breeding range is also necessary for delineating the location and breadth of a hypothesized migratory divide between myrtle warblers wintering on the Pacific Coast and those wintering on the Gulf Coast. Finally, although stable isotopes from feathers can be used to infer breeding and nonbreeding areas [37, 38], these location estimates are often broad due to the natural geographical patterns of isotope distribution, limiting the ability to link precise breeding and nonbreeding areas at a fine scale. Tracking myrtle warblers using geolocators can provide narrower estimates of nonbreeding areas as well as detailed information about individual migration routes.

Here, we use multi-sensor geolocators—sensors that measure both light levels and barometric pressure—to characterize the migrations of myrtle warblers breeding in Anchorage, Alaska at a fine scale to determine whether distance minimization or historical contingency has played a stronger role in shaping migration routes for high-latitude populations of this species. In addition, we use stable hydrogen isotopes to infer the nonbreeding areas of myrtle warblers breeding in Alaska, northern British Columbia, and Alberta to determine whether a migratory divide is present in this region between birds migrating east to the Gulf Coast and west to the Pacific Coast. If the migration routes of high-latitude breeding myrtle warblers evolved to minimize distance in order to reduce energy expenditure, we predict that myrtle warblers breeding in Alaska will migrate to the closer nonbreeding area on the Pacific Coast. However, if migration routes in this species have been constrained by historical contingency, we predict that Alaskan myrtle warblers will migrate eastward across the boreal forest to winter on the Gulf Coast. This route would reflect the likely path of past range expansion northward and westward out of a refugium in the southeastern United States, which has been inferred from genetic analyses of yellow-rumped warbler subspecies [34, 35] and paleodistribution modeling for warblers with overlapping habitat requirements [27, 28].

To characterize migration routes and test these hypotheses, we use newly developed methods to infer migration trajectories using atmospheric pressure data [36–38]. We compare the quality of tracks incorporating pressure versus those based only on traditional light-level data. This pressure data allows us to not only address hypotheses about the evolution of migration routes and the relationship between migration and geographic range, but also to describe migration timing, stopover strategies, and flight altitude—which is currently unknown for this and most other North American migratory bird species.

## Methods

### Geolocator deployment and sample collection

From June 1–14, 2022, we captured 55 myrtle warblers using song playback and mist nets in Far North Bicentennial Park and the BLM Campbell Tract, a ~ 19 km^2^ forested area located in Anchorage, Alaska (Supplementary Figure S1 and Supplementary Dataset 1). We banded and measured each bird and collected two greater covert feathers for stable isotope analysis. Because song playback primarily attracts territorial males, all but one of the captured birds were males. We attached a multi-sensor geolocator (Migrate Tech model BARP30Z11-DIP; 0.45 g) that measures ambient light intensity, temperature, and atmospheric pressure to 30 of these birds using a modified leg-loop harness designed for small songbirds [39]. Geolocators sampled light intensity every minute, recording the maximum intensity every 5 min, and recorded pressure and temperature every 20 min. We used a geolocator model without an accelerometer to ensure that the weight of the tag was no more than 3–4% of the mass of the bird. Though not recording activity data means that some ground-level flights may not be detected, we expect most migratory flights to occur at increased altitudes in this species. In addition to an aluminum USGS band, birds fitted with geolocators were given a red color band to aid in resighting the following year. To serve as controls when testing for an effect of geolocator on return rate (as, to our knowledge, geolocators have not been used before on this species), 25 birds were processed identically but not given a geolocator and were fitted with a yellow color band.

To recapture geolocator-tagged birds, we surveyed the study area from June 1–14, 2023, listening for singing myrtle warblers and playing song audio at each previous capture location and throughout the park. We resighted and recaptured 6 of 30 birds with geolocators (20%). All units recorded data for the entire deployment period. We resighted 6 of 25 color-banded control birds (24%) and recaptured two. All recaptured birds were confirmed to be the same birds breeding on the respective territory in 2022. We compared return rates of geolocator-tagged birds with control birds using a Fisher’s exact test and found no significant difference in returns (*P* = 0.75).

### Estimation of migration routes from multi-sensor geolocators

We estimated locations from the geolocator data using multiple approaches to compare their efficacy: (1) using light intensity data alone largely following the threshold method pipeline described by [40], and (2) using the R package GeoPressureR [36, 37, 41] and atmospheric pressure data either alone, or in combination with light data. The Supplementary Methods contain a detailed description of these analyses.

For the light-only approach, we annotated twilight times using the R package TwGeos [42] and performed in-habitat calibration with the R package GeoLight [43] using a subset of data from three weeks after deployment, when the birds were stationary at their breeding sites (Supplementary Table S1). We estimated locations and inferred stationary periods and timing of migratory flights using GeoLight. We discarded location estimates from within three weeks before or after the equinoxes, as similar day length worldwide during these periods result in high latitudinal error.

We then inferred migration paths using atmospheric pressure data following the approach outlined in Nussbaumer and Nussbaumer [44]. We first used TRAINSET to annotate stationary periods and migratory flights, which were identified from sudden large drops in pressure, typically > 50 hPa and lasting > 2 h [45]. We constructed pressure likelihood maps in GeoPressureR, which are computed by comparing the pressure recorded by the geolocator with both spatial and temporal variation in pressure extracted from the ERA5-Land surface-level pressure reanalysis dataset [46]. After a good fit between the pressure timeseries recorded by the geolocator and the ERA5 dataset had been achieved for each stationary period, we modeled the full trajectory of each bird using hidden Markov models implemented in GeoPressureR [36]. For each bird we estimated one trajectory using pressure data alone, and one with a model incorporating both pressure and light data. For each trajectory we estimated the altitude of the bird during stationary periods and migratory flights in GeoPressureR.

To characterize migration timing for each individual and compare migration timing estimates between the two methods (GeoLight and GeoPressureR) we plotted migration timelines using the vistime R package [47]. We compared migration timing characteristics (duration, number of flights, length of flights, length of stationary periods) between fall and spring using t-tests or Wilcoxon rank sum tests, implemented in the R packages stats or exactRankTests, respectively [41, 48].

### Estimation of nonbreeding areas from stable isotopes

We inferred the nonbreeding areas of a larger sample of myrtle warblers using stable hydrogen isotope analysis. The ratio of hydrogen isotopes (deuterium, ^2^H, to protium, ^1^H, or δ^2^H) in a feather can be used to infer the location of a bird at a certain time because of a strong correlation between the δ^2^H of a feather and the δ^2^H of precipitation where the feather was grown [49]. Adult myrtle warblers undergo both a complete molt on the breeding ground and a partial molt on the nonbreeding ground, so both locations can be inferred using stable isotopes from different generations of feathers on the same bird [31, 50, 51]. From each bird we collected the two greater coverts surrounding the molt limit: one grown during the first or definitive cycle prealternate molt (i.e., on the nonbreeding grounds; FPA/DPA) and one grown during the first preformative molt (FPF) for second-year (SY) birds or definitive prebasic molt (DPB) for after-second-year (ASY) birds (i.e., on the previous year’s breeding grounds; Supplementary Figure S2). We measured δ^2^H of alternate feathers for 167 myrtle warblers breeding in Alaska (*n* = 46), British Columbia (*n* = 54), and Alberta (*n* = 67; Fig. 1). We also assessed δ^2^H in a small subset of formative and definitive basic feathers from myrtle warblers breeding in Alaska (*n* = 12) and British Columbia (*n* = 12) to assess how well the location of feather origin inferred using stable isotopes matched known breeding locations (See Supplementary Methods). Feather sample preparation and hydrogen pyrolysis were performed at the Cornell University Stable Isotope Laboratory (Supplementary Table S4).

Based on precipitation δ^2^H variation, we expect feathers from myrtle warblers wintering on the Pacific Coast to have lower δ^2^H values (Supplementary Figure S3). To test the hypothesis that a migratory divide exists between myrtle warblers breeding in Alaska and those breeding further east, we performed a linear regression in R with longitude of the breeding site as the predictor and δ^2^H of the alternate feather as the dependent variable. To assess whether basic feathers exhibited expected δ^2^H differences based on sample collection locations, we performed a Wilcoxon rank sum test in R to test for a significant difference in δ^2^H between feathers collected in Alaska versus British Columbia (Supplementary Methods).

We estimated the most likely area of origin for each feather sample using the R package assignR [52], first calibrating the precipitation hydrogen isoscape using known-origin isotope data from 313 feather samples of 19 parulid warbler species (Supplementary Figure S4; [49, 53, 54]), and then producing calibrated isoscapes for each analyzed feather sample. To assess whether each alternate feather was more likely to have been grown on the eastern or western nonbreeding ground, we implemented an odds ratio test in assignR to compare the posterior probabilities within polygons surrounding the eastern and western nonbreeding areas where myrtle warblers are most commonly found. When estimating the odds that a sample originated from the western nonbreeding ground rather than the eastern, an odds ratio > 0.75 (the ratio of the two areas) suggested a higher likelihood that the feather was grown on the West Coast.

## Results

**Fig. 2:**
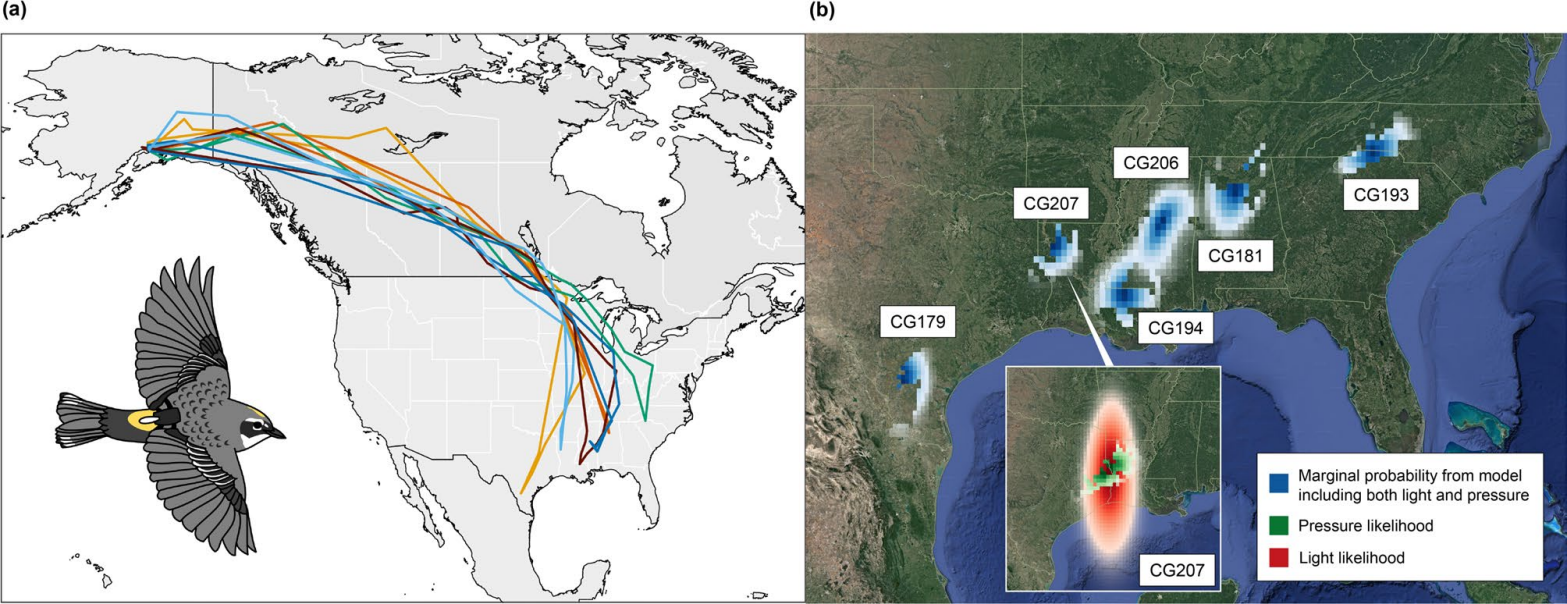
Migration routes and nonbreeding areas of geolocator-tracked myrtle warblers breeding in Alaska. (**a**) Full-year tracks inferred using combined light and atmospheric pressure data for six myrtle warblers. Each bird’s track is represented by a different color (yellow: CG179, light blue: CG207, maroon: CG194, dark blue: CG206, orange: CG181, green: CG193). (**b**) Probability of nonbreeding location for each bird produced by modeling the birds’ trajectories in GeoPressureR using both light and pressure data collected over the longest winter stationary period (blue grid cells; darker color represents higher probability). The nonbreeding area of each bird is labeled with its geolocator ID. Inset shows likelihood of nonbreeding location derived from light level data alone (red) or pressure data alone (green) for one bird. Base map: Google Satellite Hybrid (obtained through QuickMapServices QGIS plugin), retrieved 29 March 2024

All six geolocator-tracked myrtle warblers wintered in the southeastern United States, ranging from southern Texas to the Carolinas (Fig. 2). Nonbreeding areas estimated from light-level data using GeoLight largely overlapped with those inferred in GeoPressureR using either light level alone, atmospheric pressure alone, or the full model combining light, pressure, and groundspeed probability (Fig. 2b; Supplementary Figures S5 and S6). Point estimates of each bird’s most likely nonbreeding location identified using GeoPressureR versus GeoLight were highly similar (Supplementary Table S2), but showed more variation in latitude (mean difference: 1.43º, range: 0.05–4.53º) than longitude (mean difference: 0.44º, range: 0.01–0.44º).

All six birds followed largely similar routes from Alaska through Yukon and the Interior Plains of Canada, before their paths diverged after passing west of Lake Superior as each bird traveled to a different nonbreeding location in the southeastern United States (Fig. 2a; Supplementary Figures S7–S9). The birds followed similar paths for both fall and spring migration. The migration paths inferred from light data in GeoLight, from pressure data in GeoPressureR, and from the combination of light and pressure data were also similar for each bird (Supplementary Figure S10). Instances where tracks diverged from the common path through Canada or where routes estimated for the same bird using different methods differed likely resulted from uncertainty in the location estimates for certain time periods due to shading error in the light data or short pressure timeseries resulting in broad likelihood surfaces for some stationary periods.

**Fig. 3:**
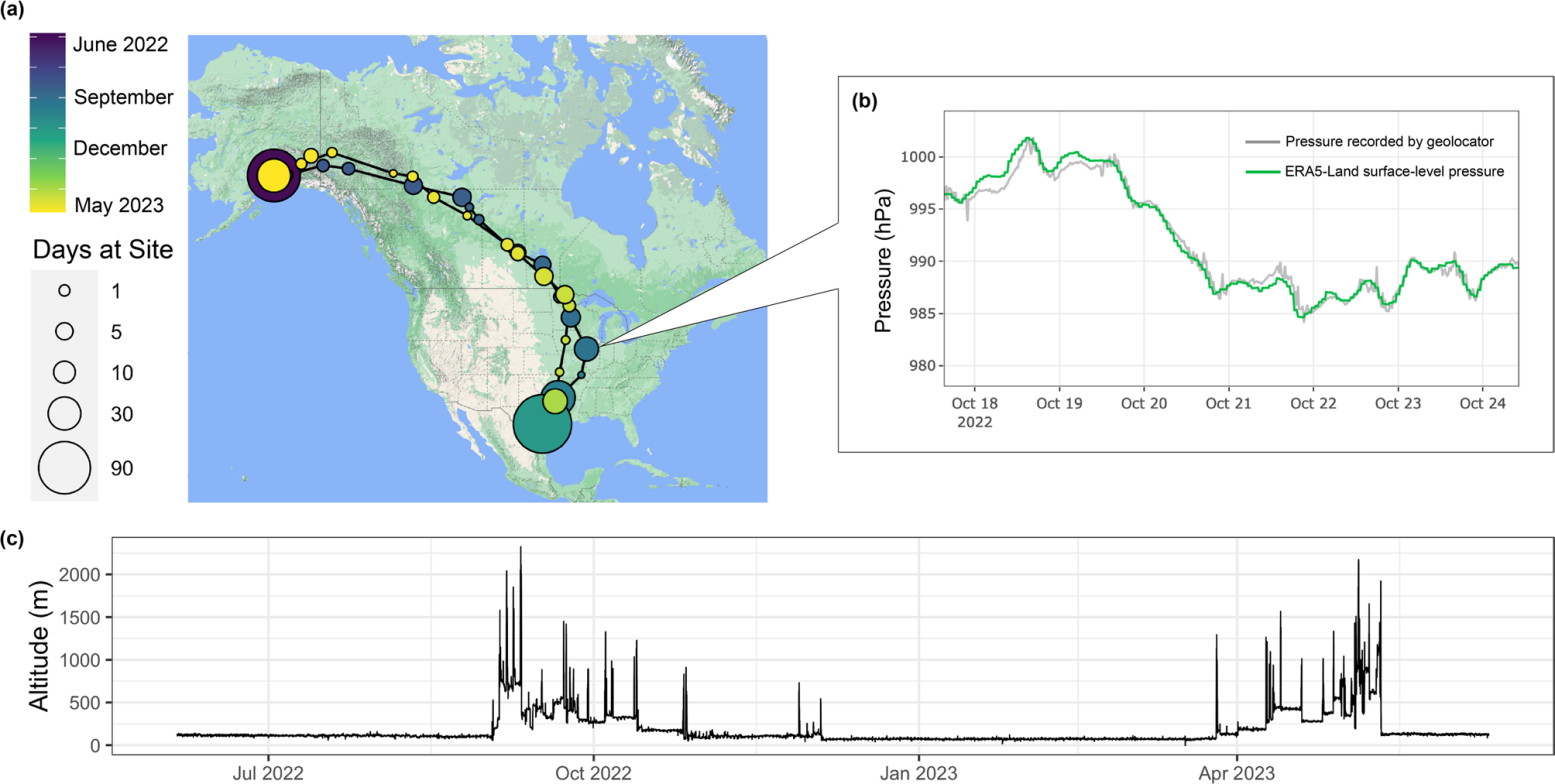
Detailed information about the migration of one geolocator-tracked myrtle warbler (CG179) derived from atmospheric pressure measurements. (**a**) Year-long migration route inferred from pressure data and movement model. The size of each circle represents the amount of time spent at a site, and color represents the time of year (fall migration: blue, spring migration: yellow). (**b**) Atmospheric pressure (hPa) measured over time by the geolocator (grey) during a stationary period versus that recorded in the ERA5-Land surface level pressure data set (green) for the same period at the inferred stopover location. (**c**) Altitude of the bird over the year estimated from the geolocator’s pressure measurements. Sudden sharp peaks in altitude occur due to migratory flights. Map data ©2024 Google, INEGI

The atmospheric pressure data allowed us to characterize migratory timing in greater detail than using light-level data alone, because each migratory flight could be identified from sharp drops in pressure (Fig. 3). By comparing the timing of these drops in pressure to the light levels recorded by the geolocator, we found that nearly all migratory flights took place in periods of darkness (Supplementary Figure S11), with the exception of some short flights that occurred during the day and very long flights that extended from night into the morning hours. The average length of a migratory flight was 6.75 h (sd = 3.41), and the longest flight was 17.7 h (Supplementary Table S3).

All six birds departed their breeding grounds in Anchorage between 28 August–5 September and arrived on their nonbreeding grounds between 29 October–4 December (Supplementary Figure S12 and Table S3). They spent between 89 and 158 days on their nonbreeding grounds, which we defined as the location of the longest winter stationary period (mean = 129, sd = 26.9). The birds departed for spring migration between 25 February–9 April and arrived back to the breeding grounds between 6 May–19 May. The mean duration of spring migration (47 days, sd = 18.9) was significantly shorter than that of fall migration (77 days, sd = 12.1; *W* = 33, *p* = 0.015), and spring migration was shorter than fall migration for all but one bird (Supplementary Figure S13 and Table S3). The birds completed spring migration in fewer flights (mean fall flights = 18, sd = 1.17; mean spring flights = 15, sd = 2.2; *W* = 32.5, *p* = 0.022; Figure S14 and Table S3), and the average length of these flights was longer in spring than fall (mean flight length per bird in fall = 5.8 h, sd = 0.48; in spring mean = 8.0 h, sd = 1.17; *t* = -4.23, df = 6.7, *p* = 0.004; Supplementary Figure S15 and Table S3). The amount of time spent at stopover sites was significantly longer in fall than spring (mean total stationary days in fall = 73, sd = 12; spring mean = 42, sd = 19; *W* = 33, *p* = 0.015; Supplementary Figure S16 and Table S3).

When comparing estimates of migration timing generated using pressure data versus light-level data, both methods identified large shifts in migratory behavior occurring at similar times (i.e. departure from the breeding ground, arrival on the nonbreeding ground; Supplementary Figure S17). However, GeoLight often split long stationary periods identified from the pressure data into multiple shorter stationary periods and merged shorter stopover periods together.

We were also able to characterize the flight altitudes of migrating myrtle warblers using atmospheric pressure data (Fig. 3, Supplementary Figures S18 and S19). The maximum altitude attained by any individual was 2903 m above sea level. The median flight altitude for each bird over the full year ranged from 721 m to 919 m a.s.l. (average = 839 m a.s.l.). Though two birds flew at significantly lower altitudes in spring compared to fall (Supplementary Figure S20), overall, there was no significant difference in median flight altitude between seasons (*t* = 1.25, *df* = 6.1, *p* = 0.26). The pressure data also allowed us to characterize finer scale vertical movements, such cyclical changes in pressure corresponding to periods of daylight and darkness when the birds were likely foraging and roosting at different elevations, and altitudinal changes occurring within a single flight (Supplementary Figure S21).

Stable isotope values (∂^2^H) of formative and basic feathers (expected to have been grown on the previous year’s breeding ground) significantly differed between samples collected in Alaska versus British Columbia (*W* = 117, *p* = 0.008; Supplementary Figure S22), with BC samples exhibiting lower ∂^2^H (mean ∂^2^H = -128) than AK samples (mean ∂^2^H = -119). The posterior probability density maps of most basic feather samples included the sampling location (breeding location in 2022) within the region of likely origin (Supplementary Figures S23 and S24), but for about half of the Anchorage-breeding birds, the posterior probability was low for the sampling site (*n* = 5; discussed in Supplementary Methods).

**Fig. 4:**
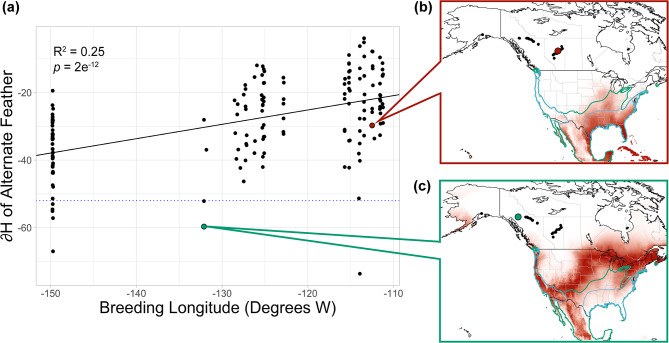
Stable hydrogen isotope analysis of alternate covert feathers sampled from myrtle warblers breeding in Alaska, British Columbia, and Alberta. (**a**) There is a significant positive relationship between hydrogen isotope ratio (∂^2^H_f_) and breeding longitude (*p* = 2e10^− 12^). Points above the dotted blue line have ∂^2^H_f_ values more suggestive of East Coast wintering, whereas those below have a higher likelihood of having been grown on the West Coast, according to an odds ratio test. Posterior probability maps are shown for (**b**) one individual that likely wintered on the East Coast and (**c**) one individual with a higher probability of wintering on the West Coast. Darker red areas represent regions of higher probability of origin for the sample. The green outline surrounds the full yellow-rumped warbler nonbreeding area, and the blue outline surround the East Coast and West Coast nonbreeding areas where myrtle warblers are most common, according to eBird abundance data. Black points indicate all locations where feathers were sampled, and larger red (**b**) or green (**c**) points show the location of the highlighted sample

There was a significant relationship between stable hydrogen isotope values (∂^2^H) in alternate feathers and breeding site longitude (β = 0.425, *df* = 165, *t* = 7.58, *p* = 2.3e^− 12^, R^2^ = 0.254; Fig. 4). When comparing the posterior probabilities of feather origin from the West Coast nonbreeding ground to the East Coast nonbreeding ground, eight samples had odds ratios greater than 0.75 (the ratio of the nonbreeding ground areas), suggesting that these birds had a higher likelihood of wintering on the Pacific Coast (Fig. 4; Supplementary Figure S25). Five of the birds with high likelihood of Pacific Coast wintering had bred in Alaska, two in British Columbia, and one in Alberta. In addition, one bird each in Alaska and Alberta had odds ratios only slightly lower than 0.75 and ∂^2^H values that were much lower than other individuals in each population. The remaining 157 birds had higher odds of wintering on the Gulf Coast, although the posterior probability density maps of many of these birds showed some high likelihood regions within the Pacific Coast nonbreeding area (Supplementary File 1).

## Discussion

We characterized the migration routes, nonbreeding locations, migration timing, and flight altitudes of six myrtle warblers in fine detail using multi-sensor geolocators and estimated nonbreeding areas for 167 individuals using stable hydrogen isotopes. Additionally, we compared methods of inferring migration routes and timing using traditional light-level data versus atmospheric pressure. Using pressure data allowed us to not only characterize migration routes with less error at finer spatial and temporal scales, but also to reveal novel details about migratory behavior, such as flight altitude. We found that, contrary to expectations based on previous studies of this system, all geolocator-tracked birds migrated to the southeastern United States rather than undergoing a shorter migration to the Pacific Coast nonbreeding ground as originally predicted. In the larger sample of birds for which we estimated nonbreeding areas using stable isotopes, most birds (95%) likely wintered in the southeast, whereas a small subset (5%) showed an increased likelihood of wintering on the Pacific Coast. Overall, these results are consistent with the “historical contingency” hypothesis, suggesting that high-latitude breeding myrtle warblers typically migrate along routes retracing historical paths of postglacial expansion rather than following the shortest, most direct route to the closest nonbreeding area.

### Most “*hooveri*”-type myrtle warblers do not take a shorter migration route to the Pacific Coast

As far back as 1899, it was thought that myrtle warblers breeding in northwestern North America—once categorized as the *S. c. hooveri* subspecies on the basis of longer wings and tails—wintered on the Pacific Coast [29, 31, 55]. Although we observed in our dataset that wings and tails were significantly longer in birds breeding farther northwest (Supplementary Figures S26 and S27), we found that nearly all of these birds wintered in the southeastern United States, regardless of size. Among birds with stable isotope data, the eight birds that most likely wintered on the Pacific Coast all had long wings and tails within the *“hooveri”* range, but nearly half of those wintering on the East Coast also exhibited this large size (Supplementary Figure S28). Therefore, size does not predict the nonbreeding area of myrtle warblers, and only a small subset of *“hooveri”*-type birds may follow the shorter western migration route. Notably, the finding that most individuals in this population migrate farther than expected is consistent with a broader pattern across avian species wherein individuals with longer, more pointed wings tend to migrate greater distances than those with shorter wings [56–58].

In addition to size, stable hydrogen isotopes from feathers of myrtle warblers migrating through Vancouver had also previously linked the Pacific Coast nonbreeding grounds with the northwestern breeding grounds [31], but sampling at a single migration site could not ascertain if a migratory divide was present on the breeding ground. By sampling feathers across an east-west transect of breeding sites from Alaska to Alberta, we determined that there is no clear migratory divide in this area between western birds migrating to the Pacific Coast and birds farther east migrating to the Gulf Coast. Instead, a small subset of birds in each sampled breeding location may migrate to the Pacific Coast, with this proportion increasing in more western populations. This pattern is similar to what has been observed in Eurasian blackcaps (*Sylvia atricapilla*), where birds breeding in continental Europe that take a recently evolved, alternate migration route to winter in Great Britain originate from across the continental breeding range, representing a low frequency phenotype in multiple breeding populations [4].

Because the same isotope ratio can indicate origin from the Pacific Coast, upper Midwest, or New England, we cannot fully rule out the possibility that birds with low ∂^2^H values actually wintered in the northeast, rather than the Pacific Coast. Myrtle warblers can be found along the New England coast in winter, according to eBird observation data (Supplementary Figure S29; [59]), but typically occur at lower frequencies compared to the Gulf Coast or Pacific Coast nonbreeding areas. Though USGS banding records contain few re-encounters of myrtle warblers banded in Alaska, all winter band recoveries occurred in the southeast (*n* = 5, Louisiana, Arkansas, and Texas) or Pacific Coast (*n* = 1, California) [60]. Future studies could analyze stable isotopes in basic feathers of myrtle warblers wintering in New England to determine if any birds breed in northwestern North America.

Future tracking or isotopic studies of myrtle warblers sampled from other parts of the northwestern breeding range or directly from the Pacific Coast nonbreeding grounds are needed to determine where most Pacific Coast-wintering birds breed. A higher proportion of Pacific Coast-wintering myrtle warblers may breed in regions north of our sampling area, as previous isotopic work on warblers migrating down the Pacific Flyway also identified regions of high probability of origin in Interior Alaska, Yukon, and the Northwest Territories [31]. Alternatively, myrtle warblers wintering on the Pacific Coast could breed in southeast Alaska, undertaking a very short-distance migration. Though this was not a region identified for birds migrating through Vancouver by Toews et al. [31], USGS banding records report one myrtle warbler banded in August on the Alaskan coast northwest of Glacier Bay that was recovered in California in November [60]. One strategy to answer this question would be to deploy geolocators on the Pacific Coast nonbreeding ground. This approach successfully identified the breeding grounds of Eurasian blackcaps wintering in Great Britain, whereas deploying geolocators on those breeding in continental Europe identified only one instance of a bird migrating to Britain [4]. However, nonbreeding site fidelity is not well studied in myrtle warblers and is likely lower than breeding site fidelity [61], which could result in fewer recovered tags.

### Myrtle warbler migration routes likely retrace historical range expansion

We found that historical contingency has likely played a stronger role than distance minimization in shaping the migration routes of myrtle warblers breeding in northwestern North America. Previous genetic work [34, 35] and paleodistribution modeling for warblers with overlapping habitat preferences [27, 28] suggest that during the LGM myrtle warblers likely occupied a refugium in the southeastern United States and subsequently expanded northward and westward, following a similar path to that observed from the geolocator tracks.

The occupancy of shared refugia and dispersal along common expansion routes during and after the LGM have given rise to geographic patterns of speciation and hybridization that coincide between many species [62, 63]. Glaciation dynamics may also have shaped migration routes in common across species that shared refugia. Notably, the myrtle warblers tracked in this study followed remarkably similar routes to those taken by other species tracked from Alaska, including tree swallows (*Tachycineta bicolor*; [25]) and the congeneric blackpoll warbler (*Setophaga striata*; [26]). Similarly, in *Catharus* thrushes, certain populations breeding in northwestern North America migrate eastward rather than down the Pacific Coast—a pattern interpreted as reflecting historical paths of postglacial range expansion [3, 12, 13, 64–66]. Such similar migration routes among passerine species breeding in northwestern North America may reflect shared historical processes shaping present-day migratory movements.

Though the results of this study suggest that postglacial range expansion played a strong role in shaping the migration routes of northwestern myrtle warblers, the “historical contingency” and “optimization” hypotheses are not mutually exclusive, and both factors may have influenced contemporary migration patterns to varying degrees. We make the assumption that the shorter migration route to the Pacific Coast is less energetically costly, as the energetic cost of migration is often assumed to scale linearly with distance traveled (e.g [67]). However, other variables such as wind direction, geographical barriers, and quality of stopover habitat also influence the metabolic cost of a particular route. Future studies that model the energetic costs of alternate migration routes or directly estimate energy consumption in migrating birds using implantable biologgers (e.g [68–70]), could clarify whether factors like wind or stopover habitat increase the energetic favorability of the eastern migration route followed by many northwestern-breeding birds.

Regardless of the precise energetic cost of each migration route, we found that most myrtle warblers in this study traveled much farther than previously thought, as they are typically characterized as “short-distance migrants” [33, 71]. Rather than following the shortest route to the Pacific Coast nonbreeding area, most myrtle warblers breeding in Anchorage traveled over 11,000 km round-trip to winter on the Gulf Coast, which is longer than the migration distances of many species considered “long-distance migrants” in other studies. Based on this result, we emphasize the importance of considering intraspecific differences in migration distance in comparative studies, and the need for more studies characterizing population-level variation in migration routes, nonbreeding areas, and migration distance.

In addition to historical contingency and energetic optimization, other mechanisms may shape migration patterns, such as fitness consequences associated with the nonbreeding grounds. Research has shown that habitat quality can impart carry-over effects on breeding success and survival in many species [7, 72], and some studies have linked longer migration distances with higher annual survival [33, 73, 74]. Greater survival and subsequent reproductive success for myrtle warblers wintering on the Gulf Coast could offset the risks and energetic costs associated with a longer migration. Future work collecting fitness-related data from each nonbreeding area is needed to assess the validity of this alternative hypothesis.

### Advantages of pressure geolocation

Both light-level and atmospheric pressure data produced similar broad estimates of migration routes, nonbreeding areas, and migration timing, but pressure data allowed us to characterize these attributes at a finer scale with less uncertainty, while providing additional information about flight altitude and behavior. Although spring migration routes inferred from light data were similar overall to those derived from pressure data, fall routes could not be ascertained using light because fall migration overlapped the equinox, resulting in high latitudinal error for this period. A major advantage of pressure geolocation was the ability to identify every stop made by a bird from drops in pressure during migratory flights. Using pressure, we could infer the locations of most stationary periods with high precision, though some shorter periods had broader areas of likelihood because the model is less able to constrain possible locations with short pressure timeseries. The nonbreeding areas derived from light versus pressure data largely coincided, but with greater overlap in longitude than latitude due to higher latitudinal error in light-level data [75]. The nonbreeding areas inferred using GeoLight showed much higher uncertainty (i.e., a wider spread of location estimates) because of shading-induced error and lower resolution for estimating the timing of when birds arrived and departed from the nonbreeding ground.

In addition to improving location and timing estimates, pressure data also provided insights into aspects of myrtle warbler behavior that could not be studied using traditional light-level geolocators. From pressure readings taken during migratory flights, we found that the birds flew at a median altitude of ~ 840 m a.s.l., reaching a maximum altitude of ~ 2,900 m a.s.l., and we were able to document changes in altitude over the course of each flight. Notably, a recent study of captive myrtle warblers flying in a wind tunnel found that the birds would maintain flights in conditions equivalent to flying at ~ 2,700 m [76]. Migratory flight altitude has been estimated more often for species that breed in Europe, many of which fly at higher altitudes than the myrtle warblers in our study [77–81]. For example, the great reed warbler (*Acrocephalus arundinaceus*) flies at a median flight altitude of 1,150–1,630 m a.s.l., reaching a maximum of ~ 6,500 m a.s.l [78, 81, 82]. Few studies of migratory flight altitude have been conducted in North America, but radar [83, 84] and radio-telemetry studies [85], along with our pressure data, suggest that bird migration in North America may occur at lower altitudes than in Europe, potentially due to differences in topography, geographic barriers, or wind patterns.

Pressure geolocators can also detect fine-scale altitudinal movements. We observed cyclical pressure changes during stationary periods in some individuals, with birds spending nighttime periods 50–100 m higher than the daytime elevation. These individuals may have been traveling to a higher-elevation nocturnal roost site and foraging at a different location during the day, a behavior recorded in myrtle warblers once previously [86]. Because pressure geolocation provides fine-scale altitudinal data for individual birds, this method is highly valuable for characterizing elevational movements and flight altitude in different species and for investigating what factors, such as weather, topography, or urbanization, may affect flight altitude throughout the annual cycle.

## Conclusion

We found that most myrtle warblers breeding in the sampled region of northwestern North America migrated over 11,000 km round-trip to winter in the southeastern United States, rather than taking a shorter, alternate migration route to the Pacific Coast nonbreeding area. The route followed by geolocator-tracked birds was similar to that taken by other songbirds breeding in northwestern North America and is consistent with the previously proposed hypothesis that current migration routes reflect range expansions after the Last Glacial Maximum. Stable isotope results suggested that a small subset of birds breeding throughout the region may winter on the Pacific Coast, with no migratory divide present but the proportion of Pacific-wintering birds increasing farther west. We found that multi-sensor geolocators recording atmospheric pressure allowed us to characterize migration routes and timing at a much finer scale than traditional light-level data would allow, while also providing information on flight altitude and fine-scale elevational movements. We recommend that researchers planning geolocation studies employ tags with barometers, as the additional pressure data greatly improved our ability to characterize migration and behavior over the annual cycle for only a small increase in the cost and weight of the tag. Fine-scale tracking of individual birds from a greater diversity of species will reveal patterns of intraspecific variation in migratory behavior and help to increase understanding of how migration routes evolve.

## Supplementary Information

Below is the link to the electronic supplementary material.

Supplementary Material 1

## Data Availability

All data and code are archived on Dryad: http://datadryad.org/share/LINK_NOT_FOR_PUBLICATION/dTSa61DTnQH_KbzUucVMrt8Sr8F0PK0QoPaKgKq3Unk. Data associated with the GeoPressureR analysis are also available in the GeoLocator Data Package format on Zenodo: 10.5281/zenodo.17195973.
