# Supplementary material for: Unexpected migration patterns in a high-latitude breeding songbird: evidence from multi-sensor geolocators and isotopes
Source: Mov Ecol. 2025 Dec 20;14:4. doi: 10.1186/s40462-025-00618-6 (PMC12829184; doi:10.1186/s40462-025-00618-6)
Supplement: Supplementary file 1 — Supplementary Material 1 [file 40462_2025_618_MOESM1_ESM.docx]

Supplementary material

**Unexpected migration patterns in a high-latitude breeding songbird: Evidence from multi-sensor geolocators and isotopes**

CONTENTS

[SUPPLEMENTARY METHODS 4](#_Toc205139238)

[Geolocator deployment and sample collection 4](#_Toc205139239)

[Estimation of migration routes and nonbreeding areas from multi-sensor geolocators 5](#_Toc205139240)

[Estimation of nonbreeding areas from stable isotopes 9](#_Toc205139241)

[Validation of stable hydrogen isotope analysis 11](#_Toc205139242)

[References 13](#_Toc205139243)

[SUPPLEMENTARY FIGURES 15](#_Toc205139244)

[Figure S1. Sampling area for geolocator study. 15](#_Toc205139245)

[Figure S2. Photograph of myrtle warbler wing exhibiting clear molt limit between alternate and basic greater covert feathers. 16](#_Toc205139246)

[Figure S3. Precipitation stable hydrogen isotope values (∂^2^H) sampled from 1000 random points in the myrtle warbler eastern nonbreeding range vs. 1000 points in the western nonbreeding range. 17](#_Toc205139247)

[Figure S4. Transfer function between precipitation stable isotope values (∂^2^H_p_) and feather isotope values (∂^2^H_f_). 18](#_Toc205139248)

[Figure S5. Likelihood maps produced using GeoPressureR for the longest winter stationary period of each bird. 19](#_Toc205139249)

[Figure S6. Comparison of nonbreeding areas for six myrtle warblers estimated using three methods 20](#_Toc205139250)

[Figure S7. Location point estimates for the breeding ground, spring migration path, and nonbreeding ground inferred from geolocator light-level data using the threshold method in the GeoLight package. 21](#_Toc205139251)

[Figure S8. Year-long migration routes for six myrtle warblers inferred from pressure data alone using the GeoPressureR package. 22](#_Toc205139252)

[Figure S9. Year-long migration routes for six myrtle warblers inferred using light-level data, pressure data, and a movement model using the GeoPressureR package. 23](#_Toc205139253)

[Figure S10. Comparison of spring migration paths for six geolocator-tracked myrtle warblers estimated using three methods 24](#_Toc205139254)

[Figure S11. Nocturnal flight behavior of one geolocator-tracked myrtle warbler (CG181) over the spring migration period. 25](#_Toc205139255)

[Figure S12. Migration timelines for six geolocator-tracked myrtle warblers estimated using atmospheric pressure data. 26](#_Toc205139256)

[Figure S13. Comparison of duration of fall and spring migration in geolocator-tracked myrtle warblers. 27](#_Toc205139257)

[Figure S14. Comparison of number of flights in fall and spring migration for geolocator-tracked myrtle warblers. 28](#_Toc205139258)

[Figure S15. Comparison of length of migratory flights in fall and spring for geolocator-tracked myrtle warblers. 29](#_Toc205139259)

[Figure S16. Comparison of length of stationary periods between migratory flights in fall versus spring for geolocator-tracked myrtle warblers. 30](#_Toc205139260)

[Figure S17. Comparisons of migration timelines generated for six geolocator-tracked myrtle warblers using atmospheric pressure data in the GeoPressureR package versus light-level data in the GeoLight R package. 31](#_Toc205139261)

[Figure S18. Altitude over the full year for six myrtle warblers determined from atmospheric pressure data recorded by geolocators. 32](#_Toc205139262)

[Figure S19. Histograms depicting distributions of flight altitude for six myrtle warblers determined from atmospheric pressure data collected by geolocators. 33](#_Toc205139263)

[Figure S20. Flight altitude during fall and spring migration for six myrtle warblers determined from atmospheric pressure data collected by geolocators. 34](#_Toc205139264)

[Figure S21. Fine scale changes in altitude observed from atmospheric pressure data collected by geolocators. 35](#_Toc205139265)

[Figure S22. Comparison of stable hydrogen isotope values (∂^2^H) from basic feathers (likely grown on the previous year’s breeding ground) of myrtle warblers breeding in Anchorage, AK vs. northern British Columbia. 36](#_Toc205139266)

[Figure S23. Stable hydrogen isotope posterior probability density maps for greater covert feathers grown in the pre-basic molt (likely on the previous year’s breeding ground) of myrtle warblers breeding in Alaska. 37](#_Toc205139267)

[Figure S24. Stable hydrogen isotope posterior probability density maps for greater covert feathers grown in the pre-basic molt (likely on the previous year’s breeding ground) of myrtle warblers breeding in northern British Columbia. 38](#_Toc205139268)

[Figure S25. Odds ratios for wintering on the Pacific Coast (“western”) vs. Gulf Coast (“eastern”) nonbreeding areas for 167 myrtle warblers comparing posterior probabilities of origin for alternate covert feathers based on stable hydrogen isotopes. 39](#_Toc205139269)

[Figure S26. Relationship between wing length and breeding location for 167 myrtle warblers breeding in northwestern North America. 40](#_Toc205139270)

[Figure S27. Relationship between tail length and breeding location for 167 myrtle warblers breeding in northwestern North America. 41](#_Toc205139271)

[Figure S28. Relationships between wing and tail length and inferred nonbreeding area. 42](#_Toc205139272)

[Figure S29. Map of myrtle warbler nonbreeding areas. 43](#_Toc205139273)

[Figure S30. Light level data from one geolocator over two time periods 44](#_Toc205139274)

[Figure S31. Light level measured over the full year for each of six geolocators. 45](#_Toc205139275)

[Figure S32. Stable hydrogen isotope ratios from feathers of myrtle warblers also tracked using geolocators. 46](#_Toc205139276)

[SUPPLEMENTARY TABLES 47](#_Toc205139277)

[Table S1. Parameters used in estimation of twilight times and calibration of light-level geolocator data. 47](#_Toc205139278)

[Table S2. Point estimates for nonbreeding areas of six myrtle warblers inferred using multi-sensor geolocators. 48](#_Toc205139279)

[Table S3. Statistics describing migration timing for six myrtle warblers tracked using multi-sensor geolocators. 49](#_Toc205139280)

[Table S4. Standard information for stable hydrogen isotope analysis 50](#_Toc205139281)

# SUPPLEMENTARY METHODS

## Geolocator deployment and sample collection

Between June 1 and June 14, 2022, we captured 55 myrtle warblers using song playback and mist nets in Far North Bicentennial Park and the BLM Campbell Tract, a ≈19 km^2^ forested area located in Anchorage, Alaska (Figure S1). All captured birds were banded, measured, and two greater covert feathers were collected for stable isotope analysis. Because of song playback primarily attracting territorial males, all but one captured birds were male. We attached a multi-sensor geolocator (Migrate Tech BARP30Z11-DIP; 0.45g) that measures ambient light intensity, temperature, and air pressure to 30 of these birds using a modified leg-loop harness designed for small songbirds (Streby et al. 2015). Geolocators sampled light intensity every minute, recording the maximum intensity every 5 minutes, and recorded pressure and temperature every 20 minutes. In addition to an aluminum USGS band, birds fitted with geolocators were also given a red color band to aid in resighting the following year. To serve as controls when testing for an effect of geolocator on return rate (as, to our knowledge, geolocators have not been used before on this species), 25 birds were processed identically but not given a geolocator and were fitted with a yellow color band to facilitate resighting.

To recapture geolocator-tagged birds, we surveyed the study area from June 1 to June 14, 2023, listening for singing myrtle warblers and playing song audio at each previous capture location and throughout the trails of the park. We resighted and recaptured 6 of 30 birds with geolocators (20%). All units recorded data for the entire deployment period. We resighted 6 of 25 color-banded control birds (24%) and recaptured two. All recaptured birds were confirmed to be the same birds breeding on the respective territory in 2022. We compared return rates of geolocator-tagged birds with control birds using a Fisher’s exact test and found no significant difference in returns (*P* = 0.75).

## Estimation of migration routes and nonbreeding areas from multi-sensor geolocators

We implemented two approaches to estimate locations from the geolocator data to compare the efficacy of the methods. First, we inferred locations using light intensity data alone, largely following the threshold method pipeline described by Lisovski et al. (2020). Then, we modeled migration routes using first atmospheric pressure data alone, and then both pressure and light data together using the R package GeoPressureR (Nussbaumer et al. 2023*a*, 2023*b*).

For the light-only approach, we first annotated twilight times using the *preprocessLight* function in the R package TwGeos (Lisovski et al. 2015) using a threshold of 0.37-1 lux, depending on the tag (Table S1 and Figure S30). We removed outlier twilight times, which likely resulted from shading, using the *twilightEdit* function in TwGeos and removal criteria of window = 4, outlier.mins = 25, and stationary.mins = 20. In-habitat calibration was performed using the *getElevation* function in the GeoLight R package using a subset of the light data from three weeks after geolocator deployment, when the birds were stationary at their breeding sites (Table S1). Visual inspection of the light data using the *LightImage* function in TwGeos showed a long period of increased shading from early July to late August (Figure S31). This shading could be due to behavioral changes in the birds during molting: warblers are thought to undergo high-intensity molt, and this loss of flight feathers likely causes them to become more sedentary and shelter under vegetation during this time (Mumme et al. 2021). A second calibration was performed for this period of increased shading, and the resulting sun elevation angles were used to estimate locations during this time. Shading during migration and on the nonbreeding ground appeared similar to that observed during the first three weeks on the breeding ground, so the sun elevation angle from the first calibration period was used for the remainder of the year. Geographic locations were estimated using the simple threshold method (*coord* function) in GeoLight. We inferred stationary periods and timing of migratory flights using a changepoint model implemented by the *changeLight* function in GeoLight (quantile = 0.9, rise.prob = 0.04, set.prob = 0.05), followed by the *mergeSites2* function (distThreshold = 100, mask = land). We plotted location points for the longest stationary period during winter (“nonbreeding area”), spring migration, and breeding area (final stationary period) using the ggmap (Kahle and Wickham 2013) and ggplot2 (Wickham 2016) R packages. We removed points between three weeks before and three weeks after the fall and spring equinoxes (1 September 2022—13 October 2022 and 27 February 2023—10 April 2023), because similar day length around the world during this period results in high error for latitude estimates. We did not plot location estimates for fall migration on these maps, because this migration occurred almost entirely during the autumn equinox period for all six birds.

We then inferred migration paths and nonbreeding areas using atmospheric pressure data alone, following the approach outlined in “A User Manual for GeoPressureR” (Nussbaumer and Nussbaumer 2024). First, we annotated stationary periods and migratory flights manually in TRAINSET, a web application for labelling time series data (Kapoor et al. 2023). Migratory flights were identified from sudden large drops in pressure, typically >50hPa and lasting at least two hours. Short flights resulting in fine-scale or altitudinal movements were discarded, and extended periods differing in atmospheric pressure within a single stationary period were marked with different elevation labels. We then constructed pressure likelihood maps using the *geopressure_map* function in the GeoPressureR package. Each likelihood map is computed by comparing the pressure recorded by the geolocator with both spatial and temporal variation in pressure extracted from the ERA5-Land surface-level pressure reanalysis dataset (Muñoz Sabater 2019). First, a spatial mask is applied to filter out ERA5 grid cells where the pressure at ground level does not match the pressure recorded by the geolocator during a given stationary period. For all remaining grid cells, the normalized mean square error is calculated between the pressure timeseries recorded by the geolocator and the ERA5 timeseries for the grid cell (Nussbaumer et al. 2023*a*).

After initial construction of pressure likelihood maps, we assessed the quality of fit between recorded geolocator data and the ERA5 pressure timeseries by identifying outliers and examining histograms of error (difference between pressure recorded by the geolocator and ERA5) for each stationary period. Stationary periods exhibiting high mismatch between pressure recorded by the tag and ERA5 were probably misclassified during labelling in TRAINSET, and likely represent two different stationary periods that were erroneously combined, or a period when the bird was spending time at different elevations. Pressure timeseries where the bird was spending short times at a different altitude were either marked with an elevation label or discarded, and series where it appeared multiple stationary periods had been combined were split.

Once a good fit between the pressure timeseries recorded by the geolocator and the ERA5 dataset had been achieved for each stationary period, we modeled the full trajectory of each bird’s migration using hidden Markov models implemented in GeoPressureR (Nussbaumer et al. 2023*b*). A trellis graph was built using the *graph_create* function with a likelihood threshold of 0.99 and groundspeed threshold of 150 km/h. We set a movement model to designate the likelihood of different groundspeeds using a gamma distribution (shape = 7, scale = 7), with a fixed probability for low groundspeeds (<15 km/h) to account for frequent short-distance flights. Using this model, we computed the most likely path taken by each bird using the *graph_most_likely* function and mapped the marginal probability of each stationary period’s location using the *graph_marginal* function to assess uncertainty in location estimates.

Finally, we incorporated both the pressure and light data into a single model in GeoPressureR. Unlike the GeoLight approach, which estimates locations for each pair of twilight estimates and defines stationary periods afterwards based on changes in twilight times, GeoPressureR first aggregates the light data into stationary periods defined by the changes in pressure (i.e. migratory flights) and then estimates the location of each stationary period based on all light readings during that time (Nussbaumer et al. 2023*a*). For the light analysis, we first estimated twilights from the light data using the *twilight_create* function in GeoPressureR and manually discarded outlier twilight estimates in TRAINSET. The analysis then largely followed the pipeline used for the pressure data alone, outlined above, but in addition to computing the pressure likelihood map, we computed a light likelihood map using the *geolight_map* function. Both the pressure likelihood map and light likelihood map are then used in conjunction with the movement model when generating the most likely trajectory of the bird. We estimated the altitude of the bird during stationary periods and migratory flights using the *plot_pressurepath* function in GeoPressureR, which computes flight altitude using the barometric formula. This function corrects for natural fluctuations in barometric pressure using the ERA5 pressure data, and uses the locations estimated for the stationary periods preceding and following the flight to retrieve the ground level pressure and temperature for the region over which the bird is flying.

After migration tracks and nonbreeding areas had been estimated using the three different methods (light data alone in GeoLight, pressure data alone in GeoPressureR, and both pressure and light in GeoPressureR), for each bird we plotted the products together on the same map to compare the results. We plotted the nonbreeding areas estimated using each method using the ggmap and ggplot2 R packages, plotting the likelihood rasters for the longest winter stationary period from the GeoPressureR analysis using *geom_spatraster* and the location point estimates generated in GeoLight for wintering period using *geom_point*. We then compared migration routes estimated using the different methods by plotting the tracks generated from GeoPressureR and the location points estimated using GeoLight on the same base map.

We visualized differences in migration timing between individuals using the vistime R package to create timeline plots (Raabe 2023). We compared migration timing characteristics (duration, number of flights, length of flights, length of stationary periods) between fall and spring using t-tests or Wilcoxon rank sum tests if the data was not normal, implemented using the R packages stats or exactRankTests, respectively (Hothorn and Hornik 2001; R Core Team 2024).

## Estimation of nonbreeding areas from stable isotopes

We inferred nonbreeding areas in a larger sample of myrtle warblers breeding across northwestern North America using stable hydrogen isotope analysis. The ratio of hydrogen isotopes (deuterium, ^2^H, to protium, ^1^H, or δ^2^H) in a feather can be used to infer the location of a bird at a certain time because of a strong correlation between the δ^2^H of a feather and the δ^2^H of precipitation where the feather was grown (Hobson and Wassenaar 1997). Adult myrtle warblers undergo both a complete molt on the breeding ground and a partial molt on the nonbreeding ground, so both locations can be inferred using stable isotopes from different generations of feathers on the same bird (Toews et al. 2014, 2017; Hunt and Flaspohler 2020). We measured the stable hydrogen ratio (δ^2^H) in greater covert feathers grown during the pre-alternate molt (i.e. on nonbreeding grounds) for 167 myrtle warblers breeding in Alaska (*n* = 46), British Columbia (*n* = 54), and Alberta (*n* = 67; Figure 1). In addition, we assessed δ^2^H in feathers grown during the pre-basic molt (i.e. on breeding grounds) for 24 myrtle warblers breeding in Alaska (*n* = 12) and British Columbia (*n* = 12) to assess how well the location of feather origin inferred using stable isotopes matched the known breeding location. Alternate and basic greater covert feathers were identified based on feather wear, as alternate feathers grown more recently on the nonbreeding ground are darker black, with bright white tips, and basic feathers are duller grey with more worn and yellowed white tips (Figure S2). Feather sample preparation and hydrogen pyrolysis were conducted at the Cornell University Stable Isotope Laboratory. The hydrogen isotope ratio δ^2^H was determined by comparing the ratio of deuterium to protium in the feather sample to the ratio in established keratin standards corrected against the Vienna Standard Mean Ocean Water (VSMOW; Table S4).

We assessed the expected distributions of isotope values for the eastern and western wintering grounds by sampling the precipitation δ^2^H at random points within each range from a precipitation isoscape model produced using IsoMap (Bowen et al. 2014). Yellow-rumped warbler range shapefiles were downloaded from BirdLife International. Because these shapefiles combine the ranges of all subspecies, and higher concentrations of wintering myrtle warblers are found in a subset of the range, we modified the shapefiles in QGIS to better represent the common wintering range of myrtle warblers based on eBird observations for myrtle warblers in January-February and the eBird Status and Trends abundance map for yellow-rumped warblers (Figure S29; Fink et al. 2023). After sampling precipitation δ^2^H at 1000 random points generated inside each “common” wintering area shapefile, we found that the expected distribution of δ^2^H is significantly different between the eastern and western wintering areas (Wilcoxon rank sum test *W*=965705, *P*<2.2e^-16^; Figure S3). Based on this result, we expect feathers from birds that wintered on the Pacific Coast to have lower δ^2^H values.

To test the hypothesis that a migratory divide exists between myrtle warblers breeding in Alaska and those breeding further east, we performed a linear regression in R with longitude of the breeding site as the predictor and δ^2^H of the alternate feather as the dependent variable. To assess whether basic feathers (grown on the previous year’s breeding ground) exhibited expected differences in δ^2^H based on the birds’ known breeding locations, we performed a Wilcoxon rank sum test in R to determine if there is a significant difference in δ^2^H between basic feathers collected in Alaska versus British Columbia.

We estimated the most likely area of geographical origin for each feather sample using the R package assignR (Ma et al. 2020). First, we calibrated a global growing season precipitation hydrogen isoscape (“GlobalPrecipGS”) using known-origin isotope data from feather samples of 19 parulid warbler species (313 samples; Hobson and Wassenaar 1997, Hobson et al. 2012, Hobson and Koehler 2015) and the *calRaster* function in assignR. This regression model generated the transfer function: δ^2^H_feather_ = 0.71* δ^2^H_precipitation_ – 17.12 (R^2^ = 0.81; Figure S4). We used the *pdRaster* function in assignR to produce calibrated isoscapes for each analyzed feather sample and plotted posterior probability density maps using the R packages ggmap and ggplot2. To assess whether each alternate feather was more likely to have been grown on the eastern or western wintering ground, we used the *oddsRatio* function in assignR to compare the posterior probabilities within the polygon representing the “common” western area vs. the polygon for the “common” eastern area. When estimating the odds that a sample originated from the western wintering ground rather than the eastern wintering ground, an odds ratio higher than 0.75 (the ratio of the two areas) suggested a higher likelihood that the feather was grown on the West Coast.

## Validation of stable hydrogen isotope analysis

Inferring locations of origin using stable isotopes has low spatial resolution due to the broad distribution of isotopes across North America, but integrating isotope data with tracking, banding, and community science data can constrain the geographic assignment of samples and corroborate isotopic results (Hallworth et al. 2013; Gregory et al. 2023). To validate our isotope analysis, we compared the isotopic assignment of basic covert feathers (expected to have been grown on the prior year’s breeding ground based on previous work; Gaddis 2011; Toews et al. 2014*b*, 2014*a*) to the breeding sites where the samples were collected. Isotopic assignments matched sampling locations for all birds from British Columbia, but only 58% from Anchorage. These mismatches—all involving second-year birds—could reflect either natal dispersal (i.e., the individual hatched at a different location than it bred the following year) or calibration uncertainty in the isoscape surrounding Anchorage due to few known-origin feathers from that region (Hobson et al. 2012). Importantly, isoscape uncertainty is less likely to affect the inference of nonbreeding areas, since there are far more known-origin parulid warbler feather samples from the continental United States that were used to generate the transfer function (Hobson et al. 2012).

We additionally compared isotope probability maps with geolocator tracks for birds with both isotope and geolocator data. For all individuals with both data types (*n* = 4), the geolocator-inferred sites fell within regions of high isotopic likelihood (Supplementary Figure S32). However, Pacific Coast assignments could not be validated in this way, since no geolocator-tracked birds wintered there. While the same isotope ratio can indicate origin from the Pacific Coast, upper Midwest, or New England, based on eBird observation data myrtle warblers are less likely to winter in the northeast or upper Midwest (Supplementary Figure S5; Sullivan et al. 2009). Though USGS banding records contain few re-encounters of myrtle warblers banded in Alaska, all winter band recoveries occurred in the southeast (*n* = 5, Louisiana, Arkansas, and Texas) or Pacific Coast (*n* = 1, California; Nakash et al. 2023). However, we cannot conclusively rule out the possibility that the birds with low ∂^2^H values were among the few myrtle warblers that winter in the northeast.

Some feathers could have lower ∂^2^H values because birds molted during spring migration rather than on the nonbreeding grounds. A study on the Pacific Flyway found 40% of myrtle warblers exhibited prealternate molt during spring migration (Gaddis 2011), but whether molt migration also occurs in individuals migrating from the southeast remains unknown. While we typically collected the innermost alternate greater covert feather—grown earliest during the molt (in myrtle warblers usually beginning in February; Hunt and Flaspohler 2020)—we cannot completely rule out that this isotopic signature is the result of molt migration, and more work is needed to characterize temporal patterns of molt in yellow-rumped warblers migrating through the Mississippi Flyway.

## References

Bowen, G. J., Z. Liu, H. B. Vander Zanden, L. Zhao, and G. Takahashi. 2014. Geographic assignment with stable isotopes in IsoMAP. Methods in Ecology and Evolution 5:201–206.

Fink, D., T. Auer, A. Johnston, M. Strimas-Mackey, S. Ligocki, O. Robinson, W. Hochachka, et al. 2023. eBird Status and Trends.

Hobson, K. A., and G. Koehler. 2015. On the use of stable oxygen isotope (δ18O) measurements for tracking avian movements in North America. Ecology and Evolution 5:799–806.

Hobson, K. A., and L. I. Wassenaar. 1997. Linking breeding and wintering grounds of Neotropical migrant songbirds using stable hydrogen isotopic analysis of feathers. Oecologia 109:142–148.

Hobson, K. A., S. L. V. Wilgenburg, L. I. Wassenaar, and K. Larson. 2012. Linking hydrogen (δ2H) isotopes in feathers and precipitation: sources of variance and consequences for assignment to isoscapes. PLOS ONE 7:e35137.

Hothorn, T., and K. Hornik. 2001. exactRankTests: Exact distributions for rank and permutation tests.

Hunt, P. D., and D. J. Flaspohler. 2020. Yellow-rumped Warbler (*Setophaga coronata*), version 1.0. Birds of the World.

Kahle, D., and H. Wickham. 2013. ggmap: Spatial Visualization with ggplot2. The R Journal 5:144.

Kapoor, R., A. Pillarisetti, and Z. Shearer. 2023. TRAINSET. Github Repository.

Lisovski, S., S. Bauer, M. Briedis, S. C. Davidson, K. L. Dhanjal-Adams, M. T. Hallworth, J. Karagicheva, et al. 2020. Light-level geolocator analyses: A user’s guide. Journal of Animal Ecology 89:221–236.

Lisovski, S., M. D. Sumner, and S. J. Wotherspoon. 2015. TwGeos: Basic data processing for light based geolocation archival tags. Github Repository.

Ma, C., H. B. Vander Zanden, M. B. Wunder, and G. J. Bowen. 2020. assignR: An R package for isotope-based geographic assignment. Methods in Ecology and Evolution 11:996–1001.

Mumme, R. L., R. S. Mulvihill, and D. Norman. 2021. High-intensity flight feather molt and comparative molt ecology of warblers of eastern North America. Ornithology 138:ukaa072.

Muñoz Sabater, J. 2019. ERA5-Land hourly data from 1950 to present. Copernicus Climate Change Service (C3S) Climate Data Store (CDS).

Nussbaumer, R., M. Gravey, M. Briedis, and F. Liechti. 2023*a*. Global positioning with animal-borne pressure sensors. Methods in Ecology and Evolution 14:1104–1117.

Nussbaumer, R., M. Gravey, M. Briedis, F. Liechti, and D. Sheldon. 2023*b*. Reconstructing bird trajectories from pressure and wind data using a highly optimized hidden Markov model. Methods in Ecology and Evolution 14:1118–1129.

Nussbaumer, R., and A. Nussbaumer. 2024. GeoPressureManual: v3.0.0. [object Object].

R Core Team. 2024. R: A language and environment for statistical computing. R Foundation for Statistical Computing, Vienna, Austria.

Raabe, S. 2023. vistime: Pretty timelines in R. R Package.

Streby, H. M., T. L. McAllister, S. M. Peterson, G. R. Kramer, J. A. Lehman, and D. E. Andersen. 2015. Minimizing marker mass and handling time when attaching radio-transmitters and geolocators to small songbirds. The Condor 117:249–255.

Toews, D. P. L., J. Heavyside, and D. E. Irwin. 2017. Linking the wintering and breeding grounds of warblers along the Pacific Flyway. Ecology and Evolution 7:6649–6658.

Toews, D. P. L., M. Mandic, J. G. Richards, and D. E. Irwin. 2014. Migration, mitochondria, and the yellow-rumped warbler. Evolution 68:241–255.

Wickham, H. 2016. ggplot2. Use R! Springer International Publishing, Cham.

# SUPPLEMENTARY FIGURES

**
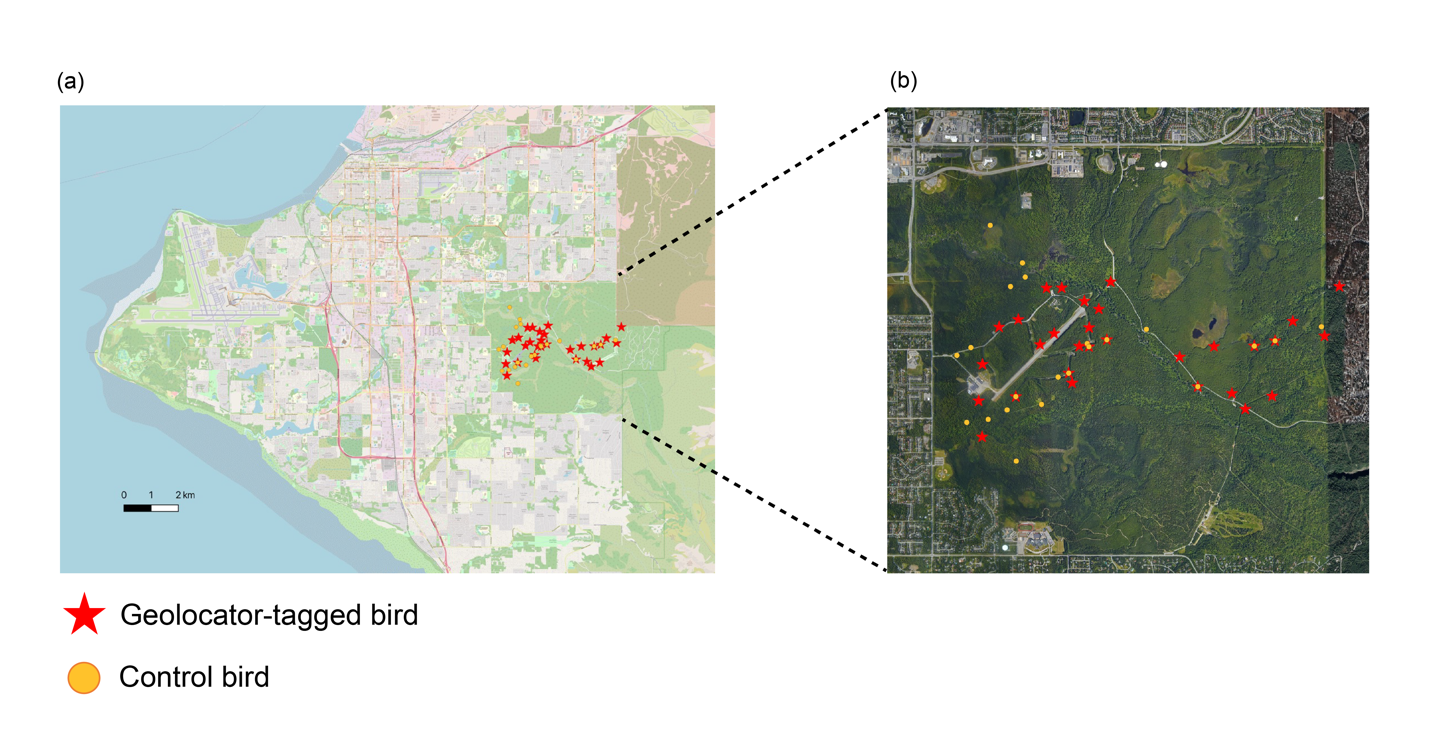
**

Figure S1. Sampling area for geolocator study. (a) Location of study area within Anchorage, AK. (b) Locations where myrtle warblers were captured within Far North Bicentennial Park. Red stars indicate sites where myrtle warblers were tagged with geolocators (*n* = 30), yellow circles represent sites where “control” birds received a color band, but not a geolocator (*n* = 25). Base map: Open Street Map Standard (obtained through QuickMapServices QGIS plugin), retrieved 22 September 2022.

**
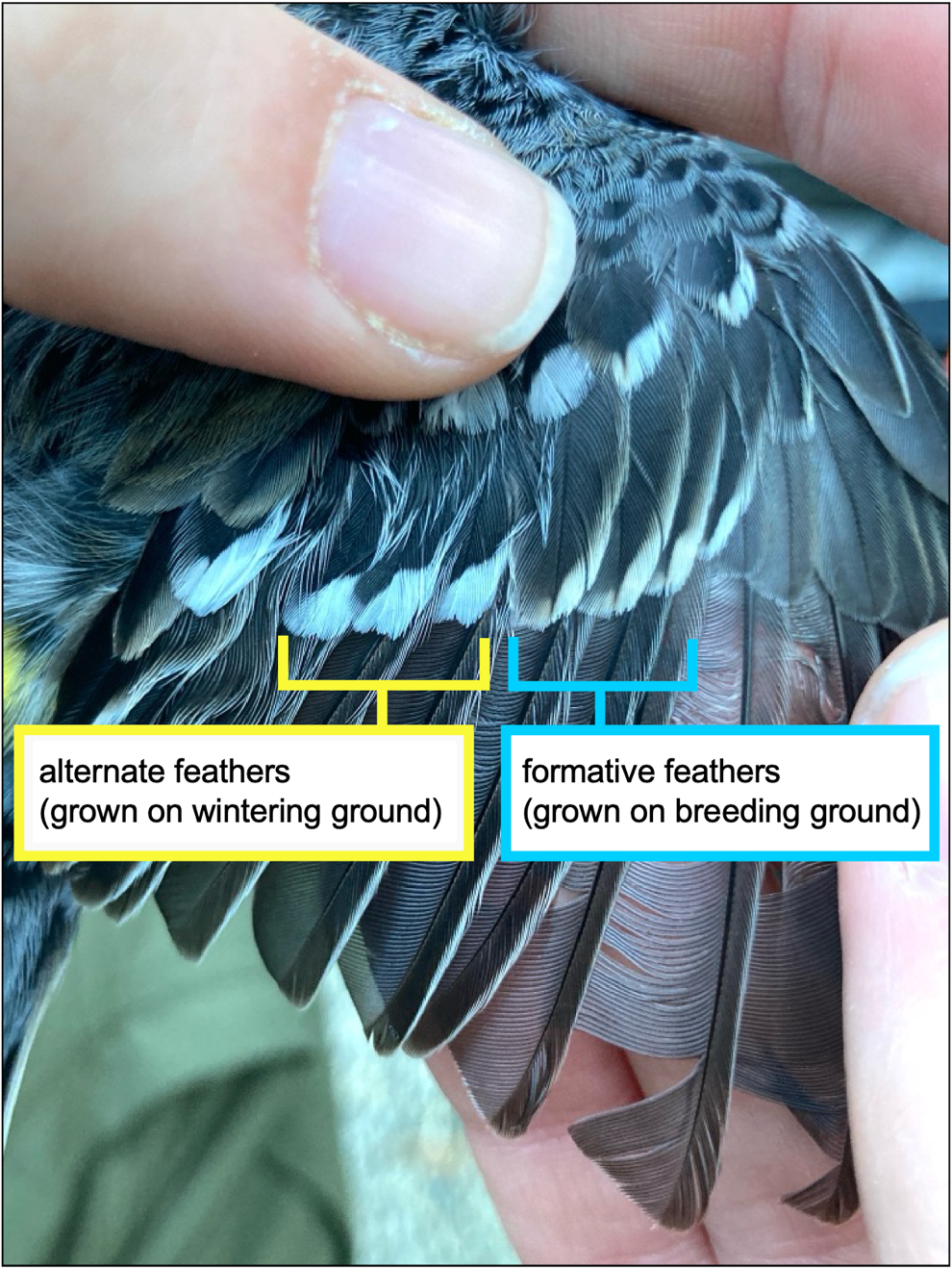
**

Figure S2. Photograph of myrtle warbler wing exhibiting clear molt limit between alternate and formative greater covert feathers. Alternate feathers were likely grown on the nonbreeding ground, and formative feathers grown on the previous year’s breeding ground. Alternate feathers, grown more recently, are darker black with brighter and more extensive white tips.


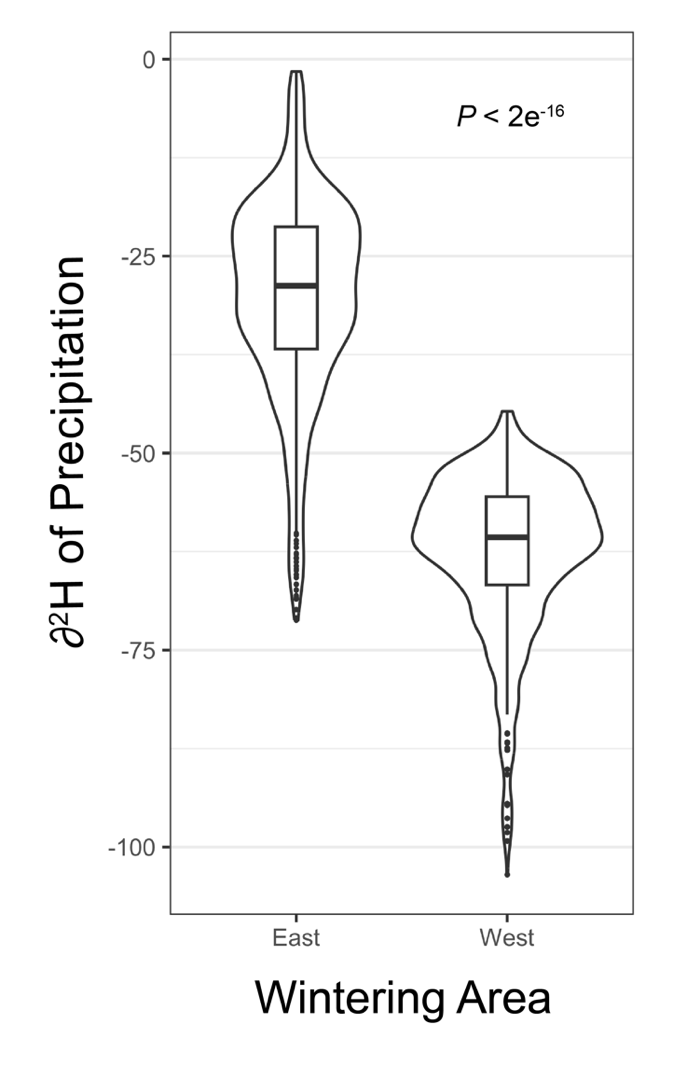


Figure S3. Precipitation stable hydrogen isotope values (∂^2^H) sampled from 1000 random points in the myrtle warbler eastern nonbreeding range vs. 1000 points in the western nonbreeding range. The western nonbreeding range exhibited significantly lower ∂^2^H values (P < 2e^-16^), so myrtle warbler feathers grown on the Pacific Coast nonbreeding ground are expected to have lower stable hydrogen ratios compared to those grown on the Gulf and Atlantic Coasts.

Figure S4. Transfer function between precipitation stable isotope values (∂^2^H_p_) and feather isotope values (∂^2^H_f_). The transfer function was generated from 313 feather samples from 19 parulid warbler species with known origins.


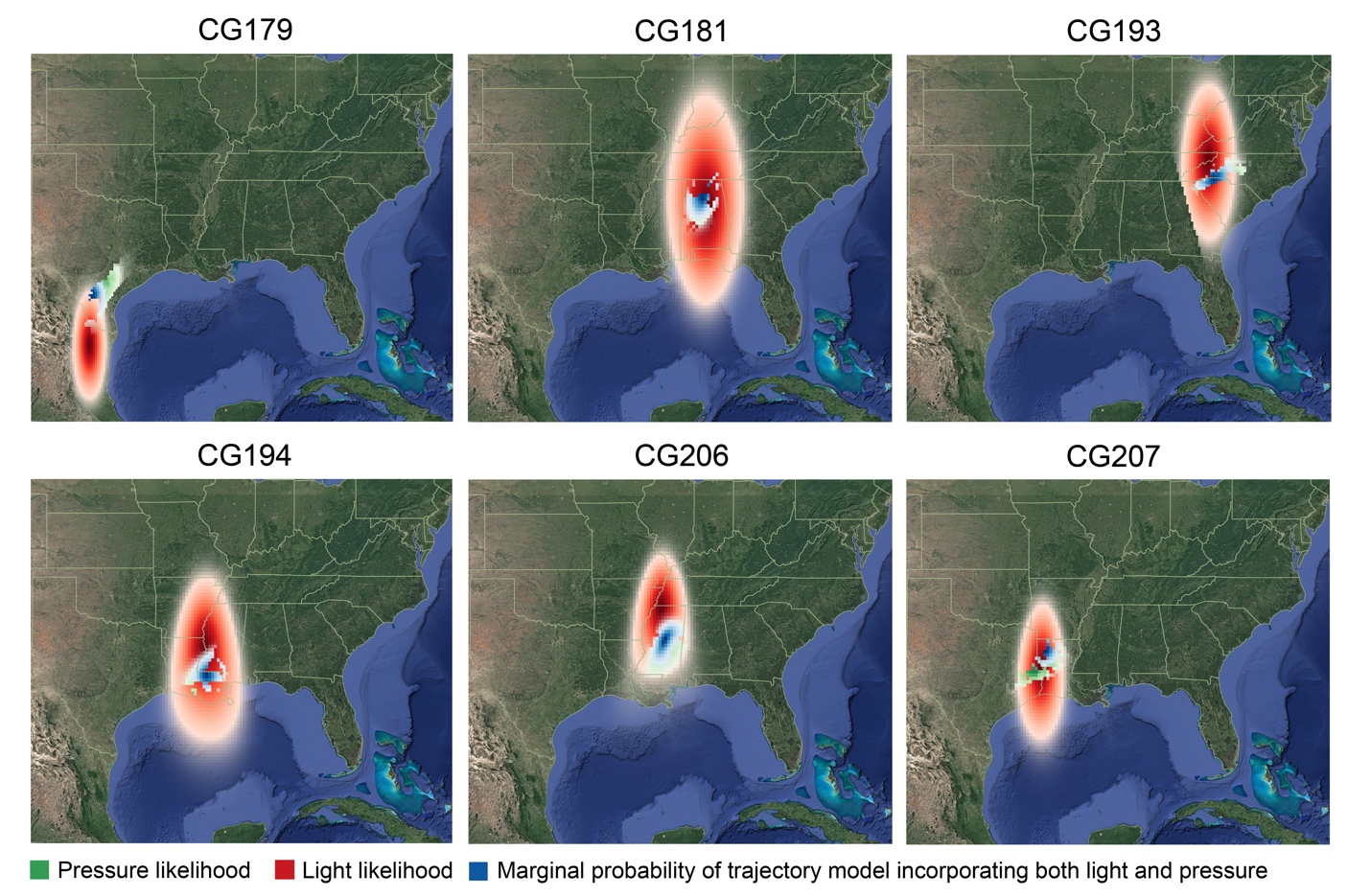


Figure S5. Likelihood maps produced using GeoPressureR for the longest winter stationary period of each bird. Red ovals represent likely nonbreeding areas based on light data alone, green pixels represent likely nonbreeding areas based on atmospheric pressure data alone, and blue pixels show the marginal probability of the model incorporating both light and pressure data. Darker colors represent higher likelihoods for all three products. For some tags (e.g. CG181, CG193, CG194) the pressure likelihood and marginal probability of the full model show high overlap, so few green pixels are visible. Base map: Google Satellite Hybrid (obtained through QuickMapServices QGIS plugin), retrieved 2 April 2024.

**
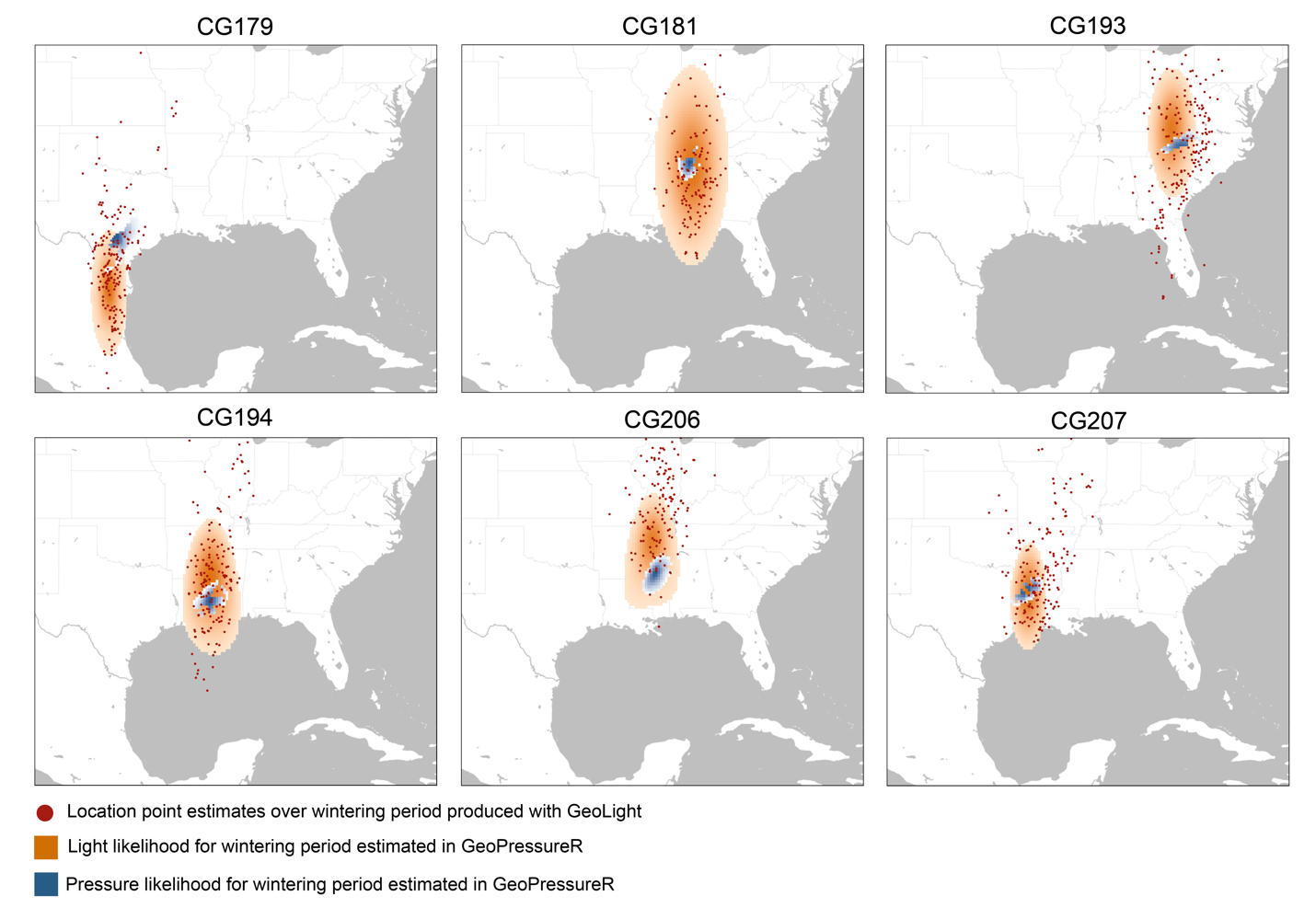
**

Figure S6. Comparison of nonbreeding areas for six myrtle warblers estimated using three methods: light level data alone analyzed using the threshold method in GeoLight (red), light level data using GeoPressureR (orange), and pressure data alone using GeoPressureR (blue). Darker colors represent higher likelihoods for all three products. Base map: Stamen Design Toner Background, data from OpenStreetMap, retrieved 3 April 2024.

**
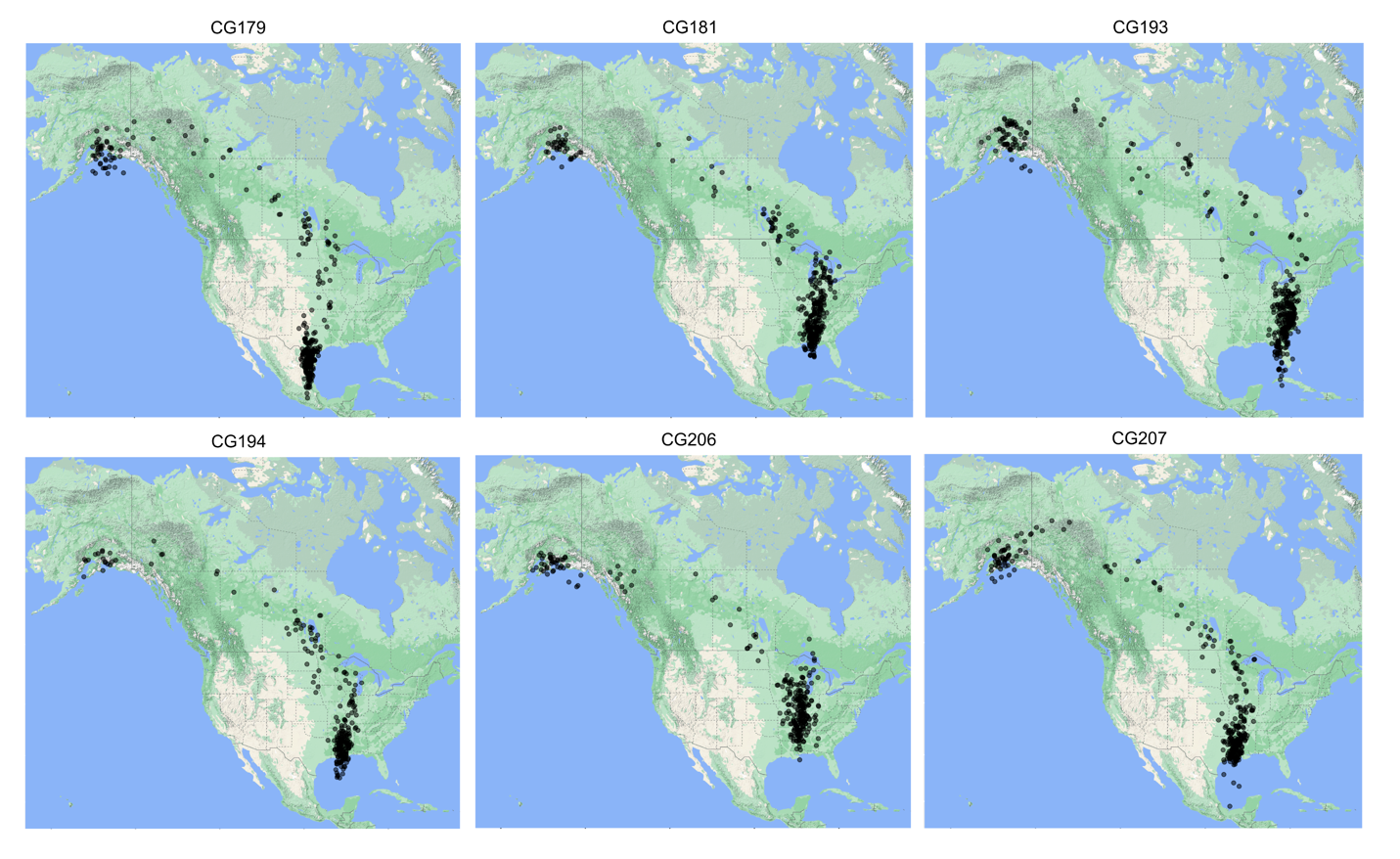
**

Figure S7. Location point estimates for the breeding ground, spring migration path, and nonbreeding ground inferred from geolocator light-level data using the threshold method in the GeoLight package. Each panel shows the track of one bird. The fall migration path was not plotted due to high error in latitude estimates resulting from equal day lengths near the autumn equinox, and points from within three weeks before and after the spring equinox were also removed for the same reason. Map data ©2024 Google, INEGI.


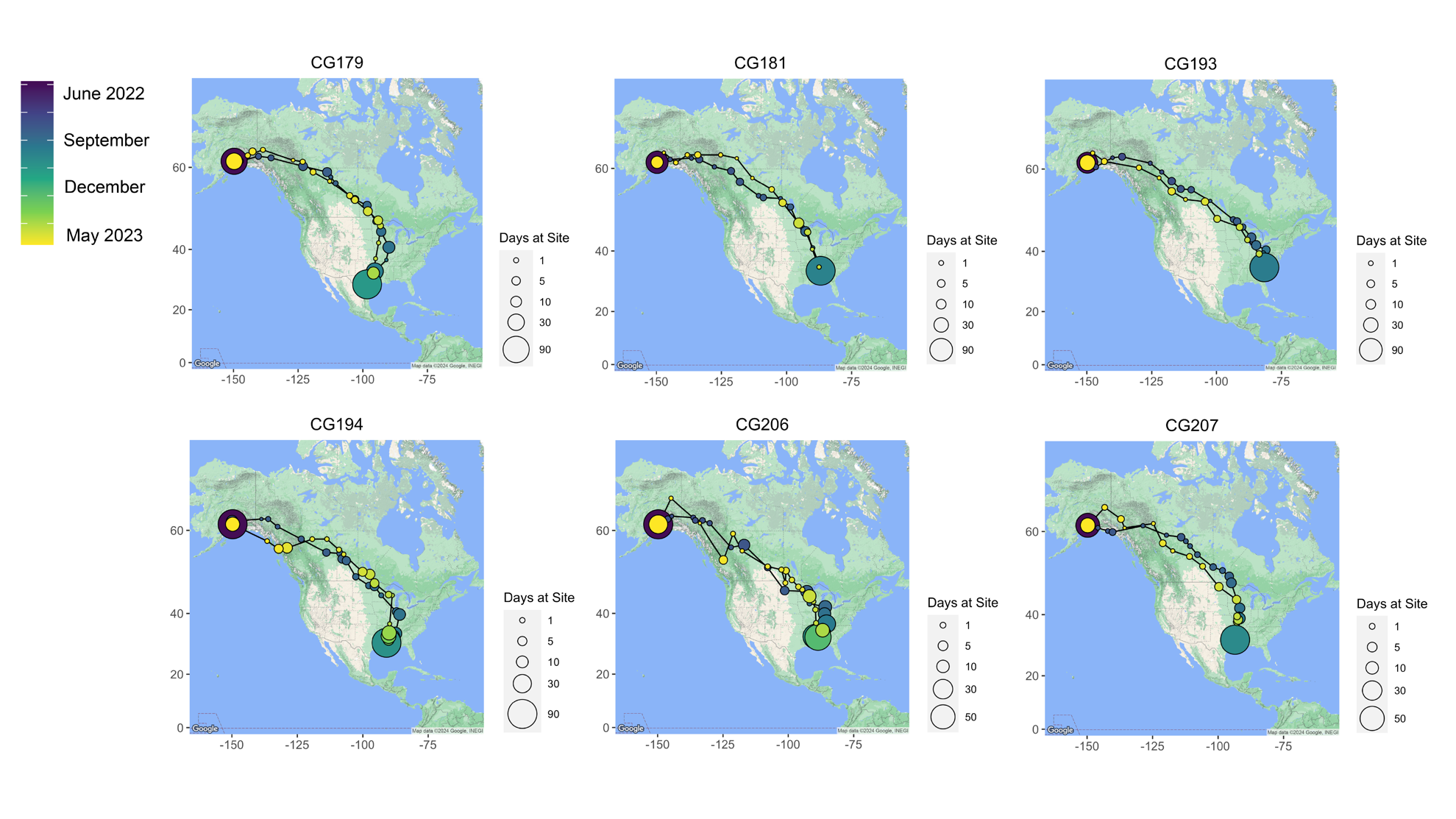


Figure S8. Year-long migration routes for six myrtle warblers inferred from pressure data alone using the GeoPressureR package. The size of each circle represents the amount of time spent at a site, and the color represents time of year (fall migration: blue, spring migration: yellow).


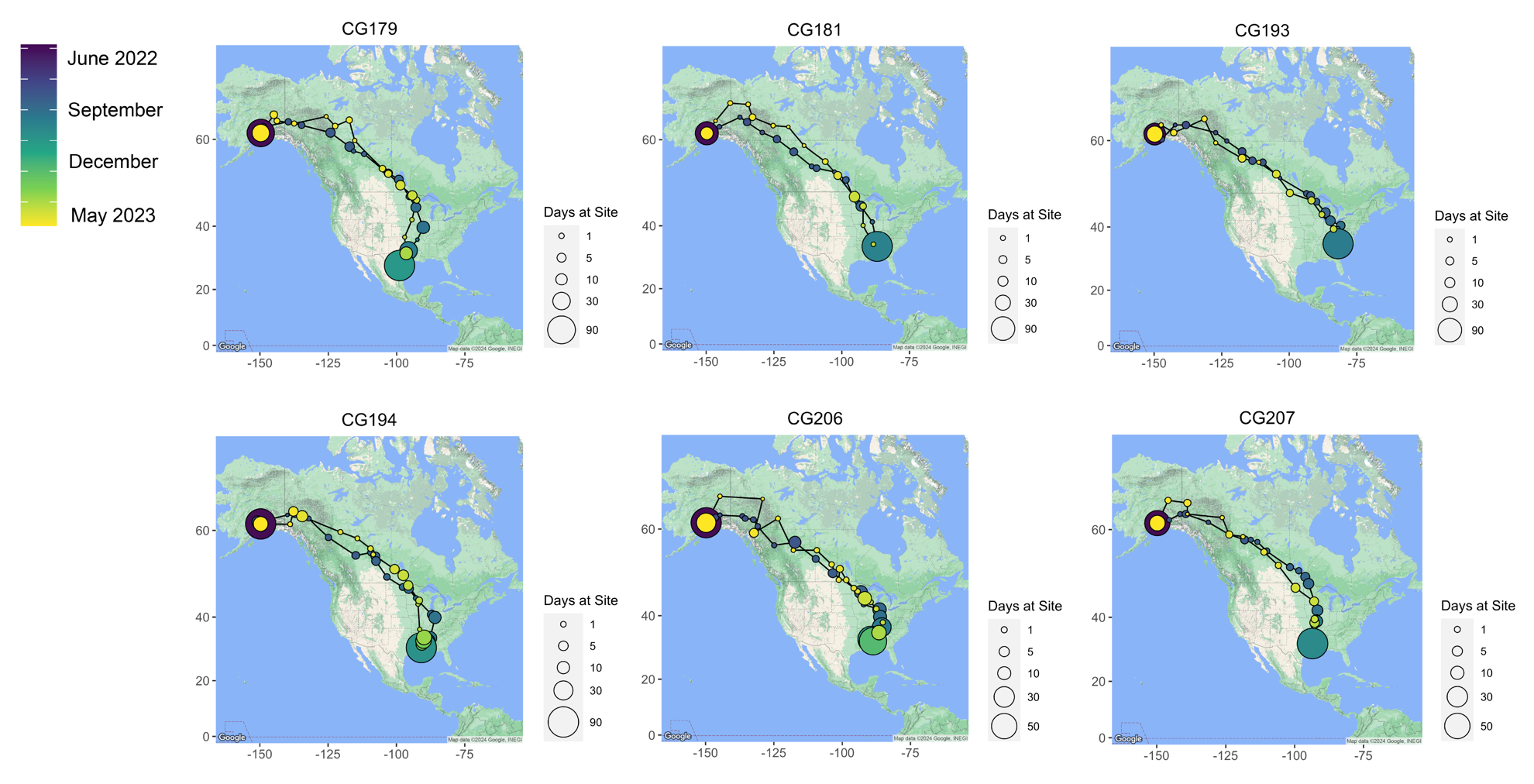


Figure S9. Year-long migration routes for six myrtle warblers inferred using light-level data, pressure data, and a movement model using the GeoPressureR package. The size of each circle represents the amount of time spent at a site, and the color represents time of year (fall migration: blue, spring migration: yellow).


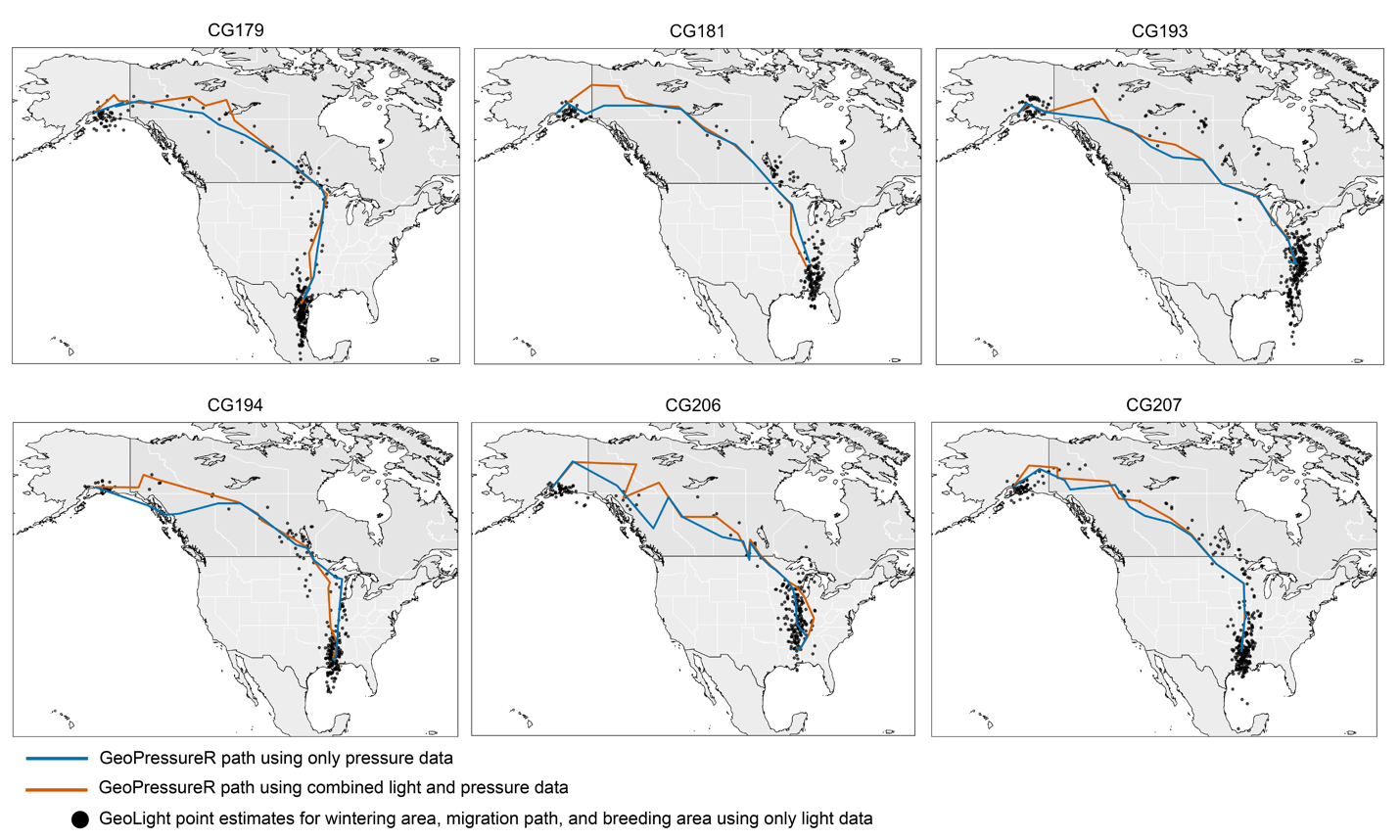


Figure S10. Comparison of spring migration paths for six geolocator-tracked myrtle warblers estimated using three methods: light level data alone analyzed using the threshold method in GeoLight (black points), pressure data alone using GeoPressureR (blue path), and a model incorporating light, pressure, and movement speed probability in GeoPressureR (orange path).


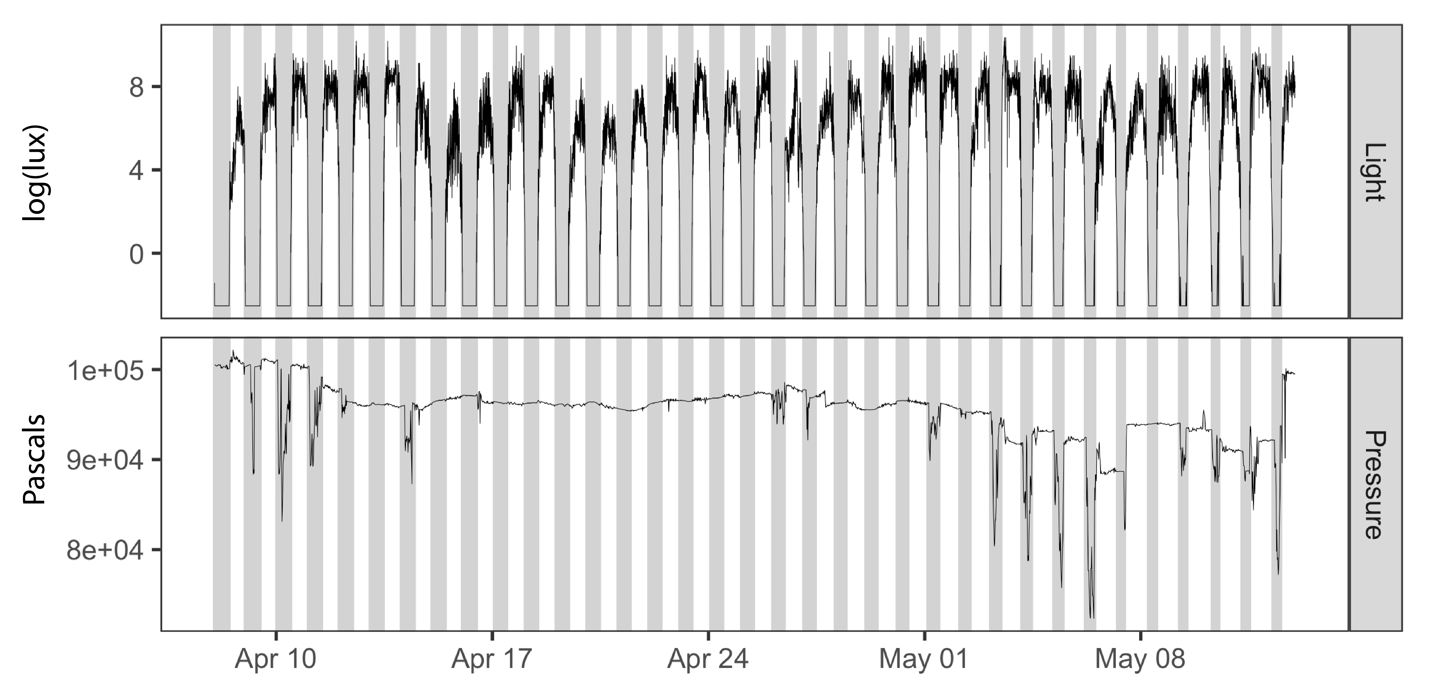


Figure S11. Nocturnal flight behavior of one geolocator-tracked myrtle warbler (CG181) over the spring migration period. Top panel shows light level measured by the geolocator (log transformed for better visualization). Bottom panel shows atmospheric pressure in Pascals for the same time period. Periods where the geolocator measured complete darkness are shaded grey. Large, sharp drops in pressure indicative of migratory flights almost always occur during periods of darkness, demonstrating the nocturnal migratory behavior of myrtle warblers.


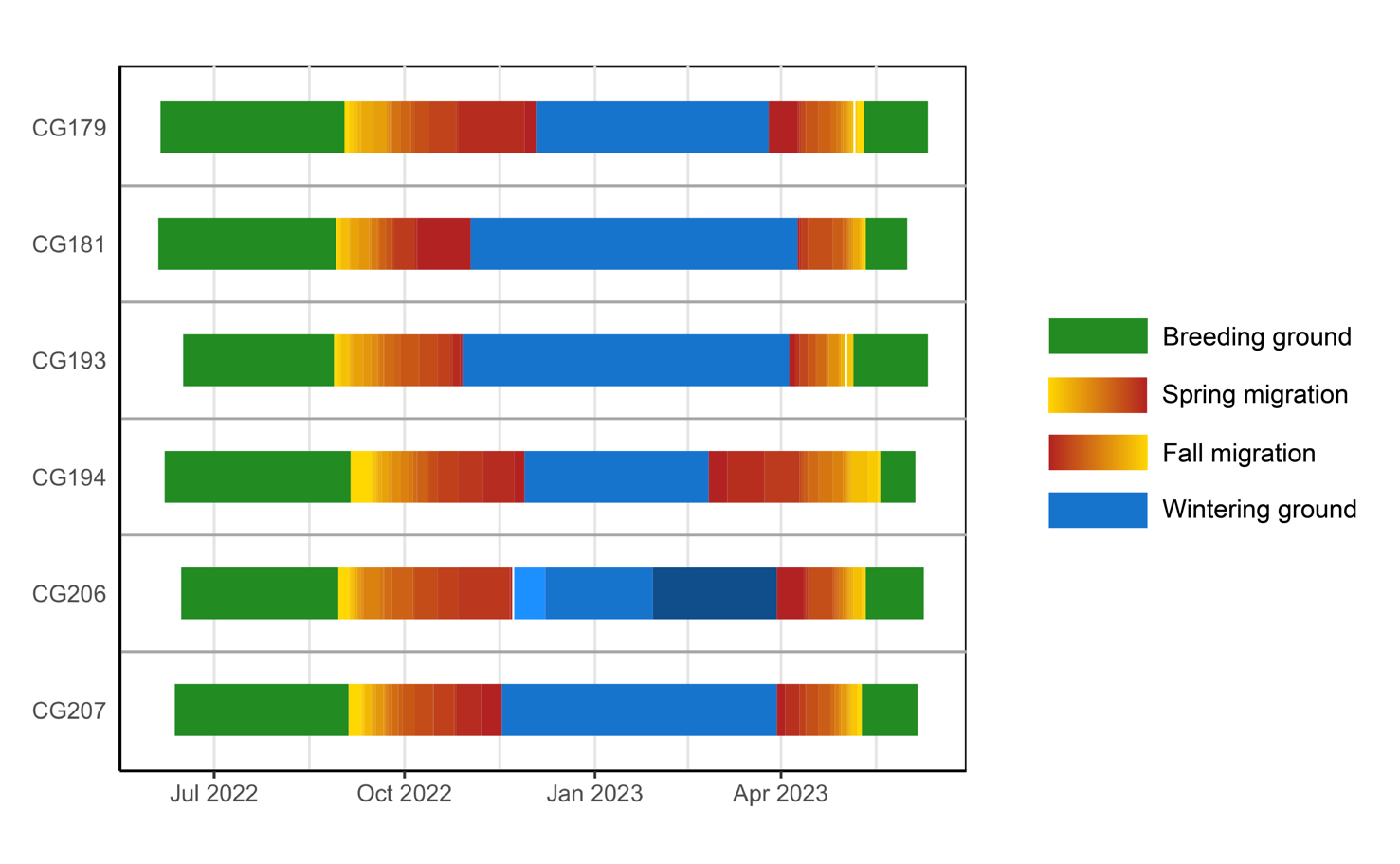


Figure S12. Migration timelines for six geolocator-tracked myrtle warblers estimated using atmospheric pressure data. Each horizontal bar represents the timeline for one bird. Color coding indicates time spent on the breeding ground (green), migrating (yellow/red gradient), and on the nonbreeding ground (blue). Bird CG206 moved between three locations within 150km during the wintering period, and time spent at each of these sites is indicated using different shades of blue.


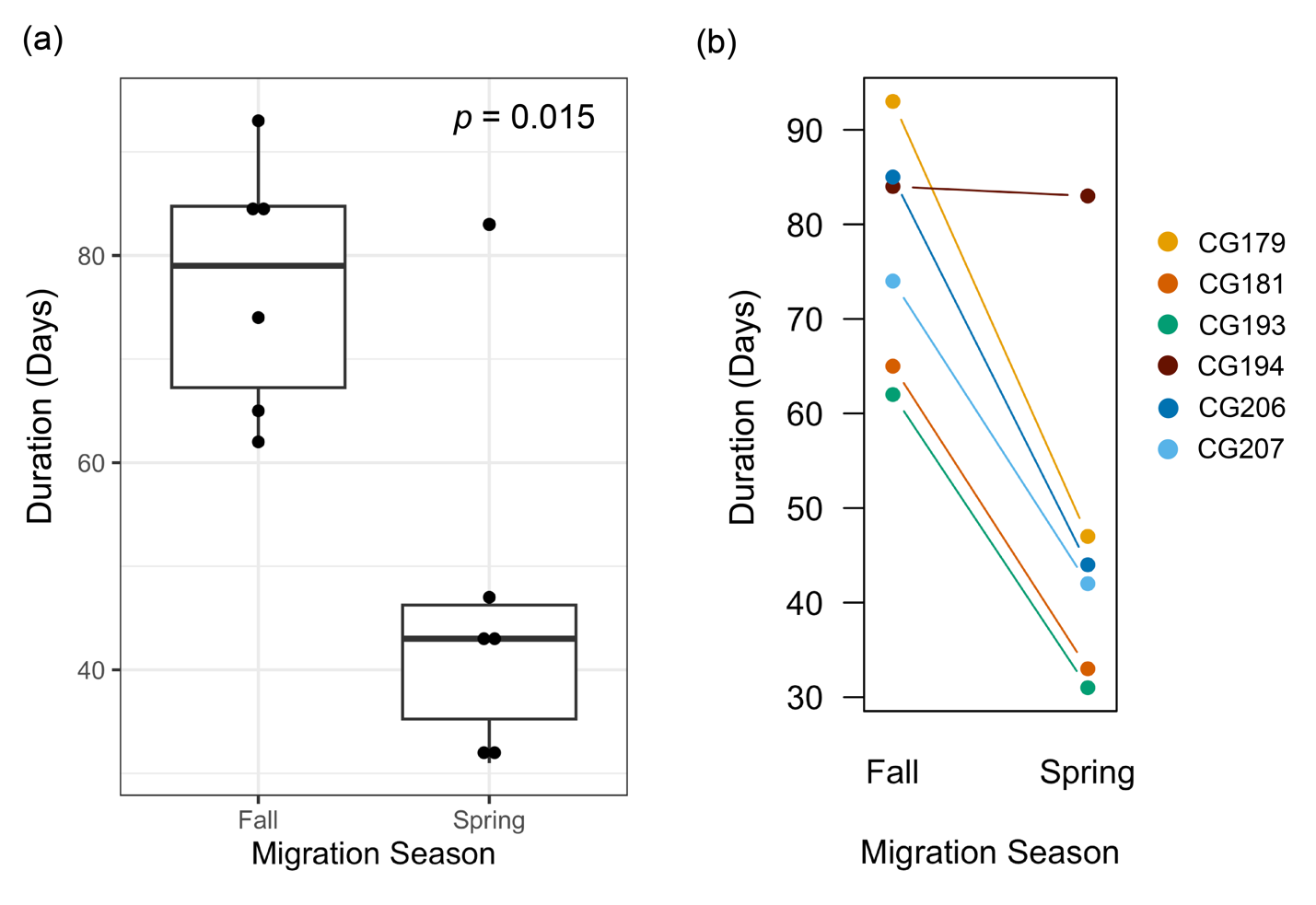


Figure S13. Comparison of duration of fall and spring migration in geolocator-tracked myrtle warblers. (a) Boxplots showing median and interquartile range of migration duration in fall and spring. Migration duration was significantly lower in spring (*W*=33, *p*=0.015). (b) Interaction plot showing differences in migration duration between seasons for individual birds.


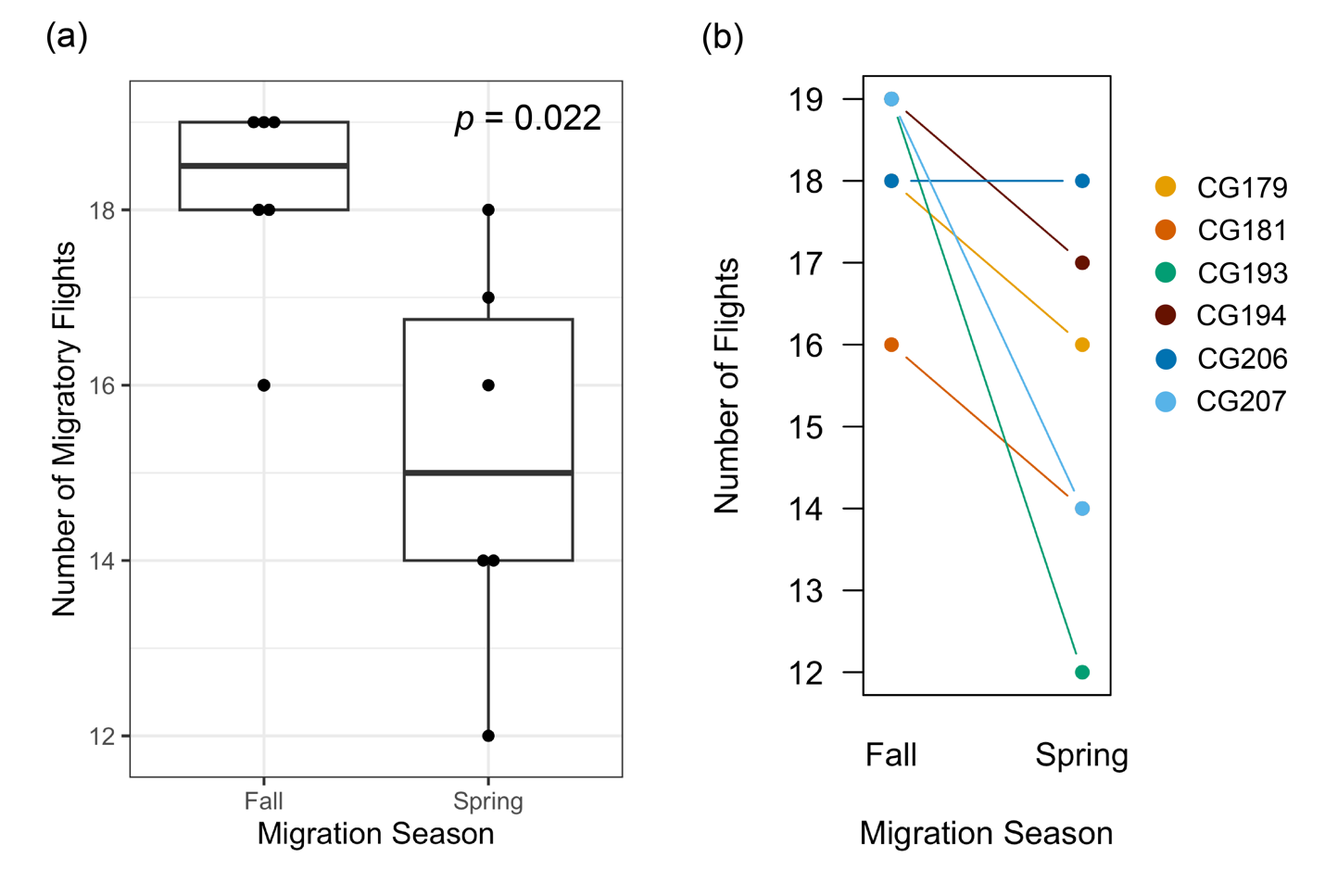


Figure S14. Comparison of number of flights in fall and spring migration for geolocator-tracked myrtle warblers. (a) Boxplots showing median and interquartile range of number of migratory flights in fall and spring. The number of flights was significantly lower in spring (*W*=32.5, *p*=0.022). (b) Interaction plot showing differences in the number of migratory flights between seasons for individual birds.


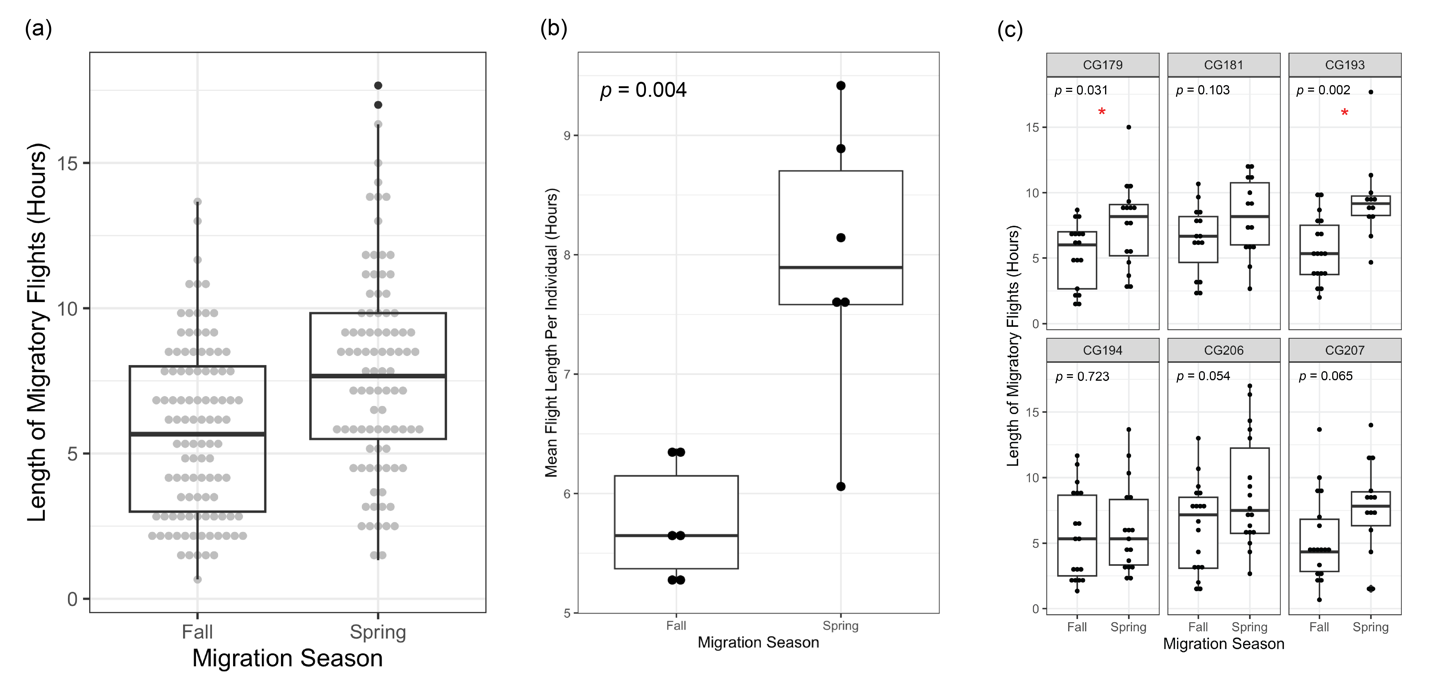


Figure S15. Comparison of length of migratory flights in fall and spring for geolocator-tracked myrtle warblers. (a) Boxplots showing length of migratory flights in fall vs. spring including all flights from all six birds. (b) Boxplots showing the mean flight length over a season for each bird. The mean flight length per bird was significantly higher in spring than fall (*t* = -4.23, df = 6.7, *p* = 0.004). (c) Boxplots showing differences in flight length between spring and fall for each individual bird.


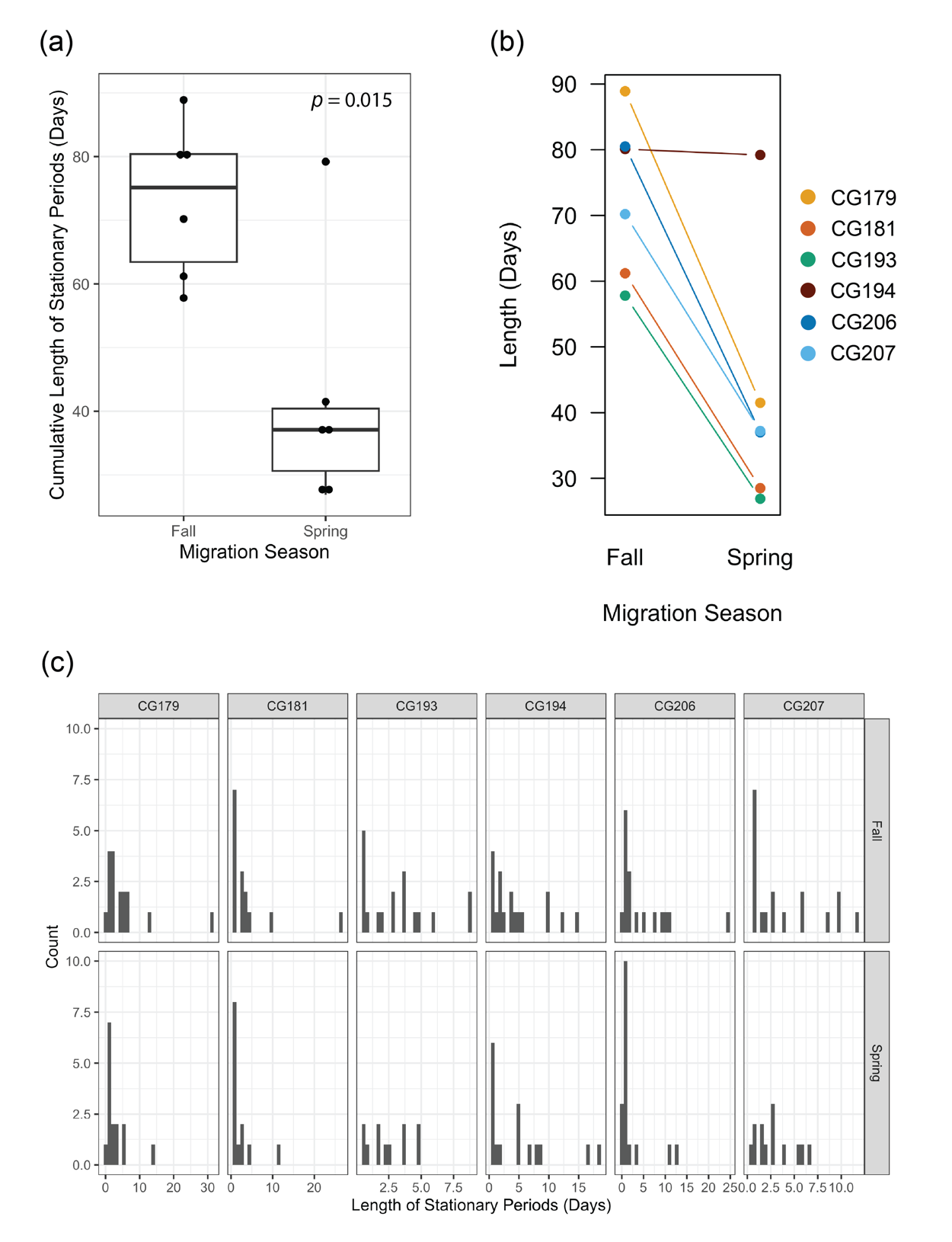


Figure S16. Comparison of length of stationary periods between migratory flights in fall versus spring for geolocator-tracked myrtle warblers. (a) Boxplots showing total stationary time during fall vs. spring migration (sum of lengths of all stationary periods during the season). Birds were stationary for significantly more time during fall than spring migration (*W* = 33, *p* = 0.015). (b) Interaction plot showing total stationary time in fall vs. spring migration for each individual bird. (c) Histograms of stationary period lengths for each bird in fall and spring migration.


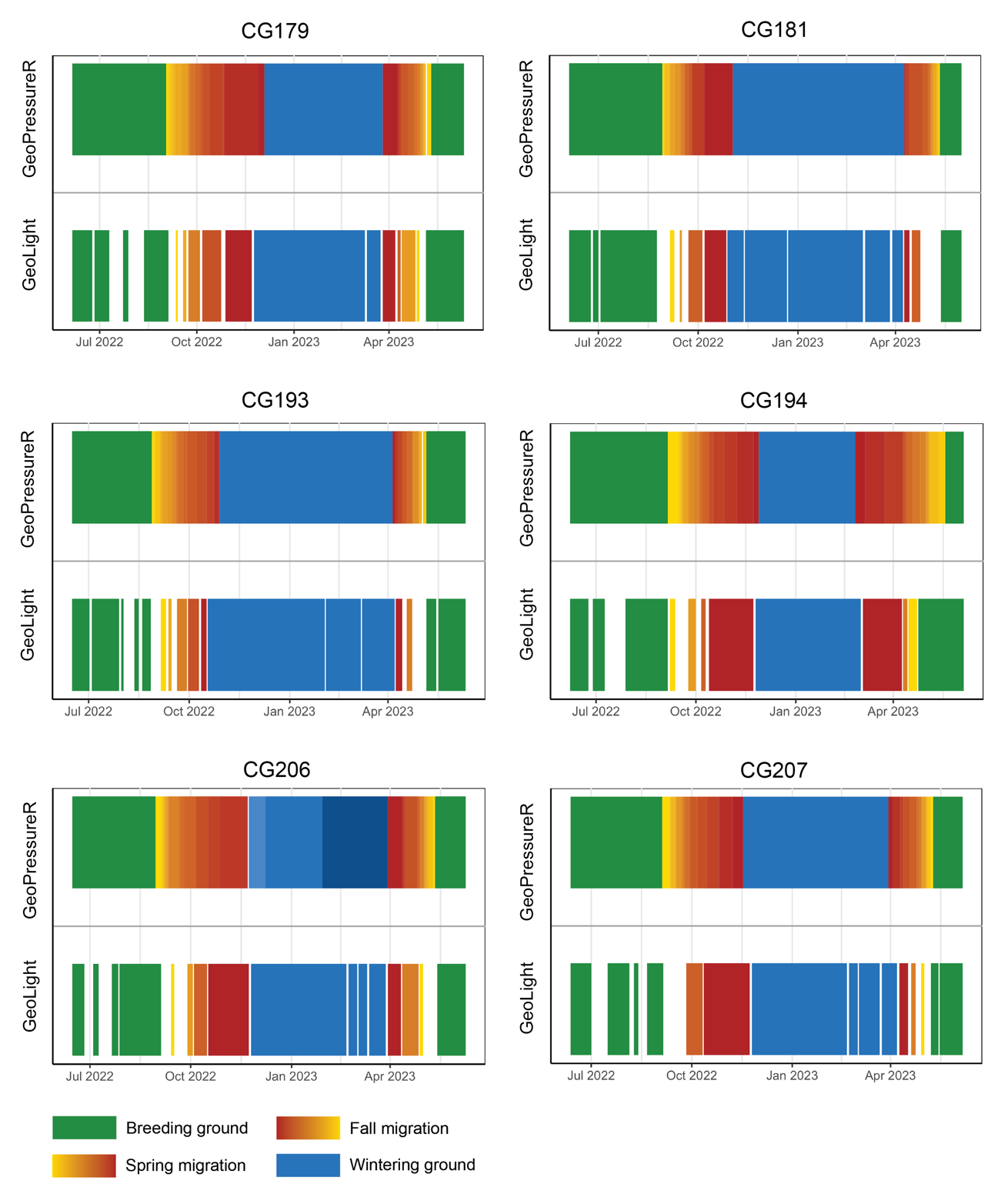


Figure S17. Comparisons of migration timelines generated for six geolocator-tracked myrtle warblers using atmospheric pressure data in the GeoPressureR package versus light-level data in the GeoLight R package. Each panel of two timelines represents data from one bird. Color coding indicates time spent on the breeding ground (green), migrating (yellow/red gradient), and on the nonbreeding ground (blue). Stationary periods estimated using GeoLight that aligned with the breeding or wintering periods identified by GeoPressureR were also colored green or blue, respectively. White spaces in the GeoLight timelines are periods when the bird was not inferred to be stationary. Bird CG206 moved between three locations within 150km during the wintering period, and time spent at each of these sites is indicated using different shades of blue.


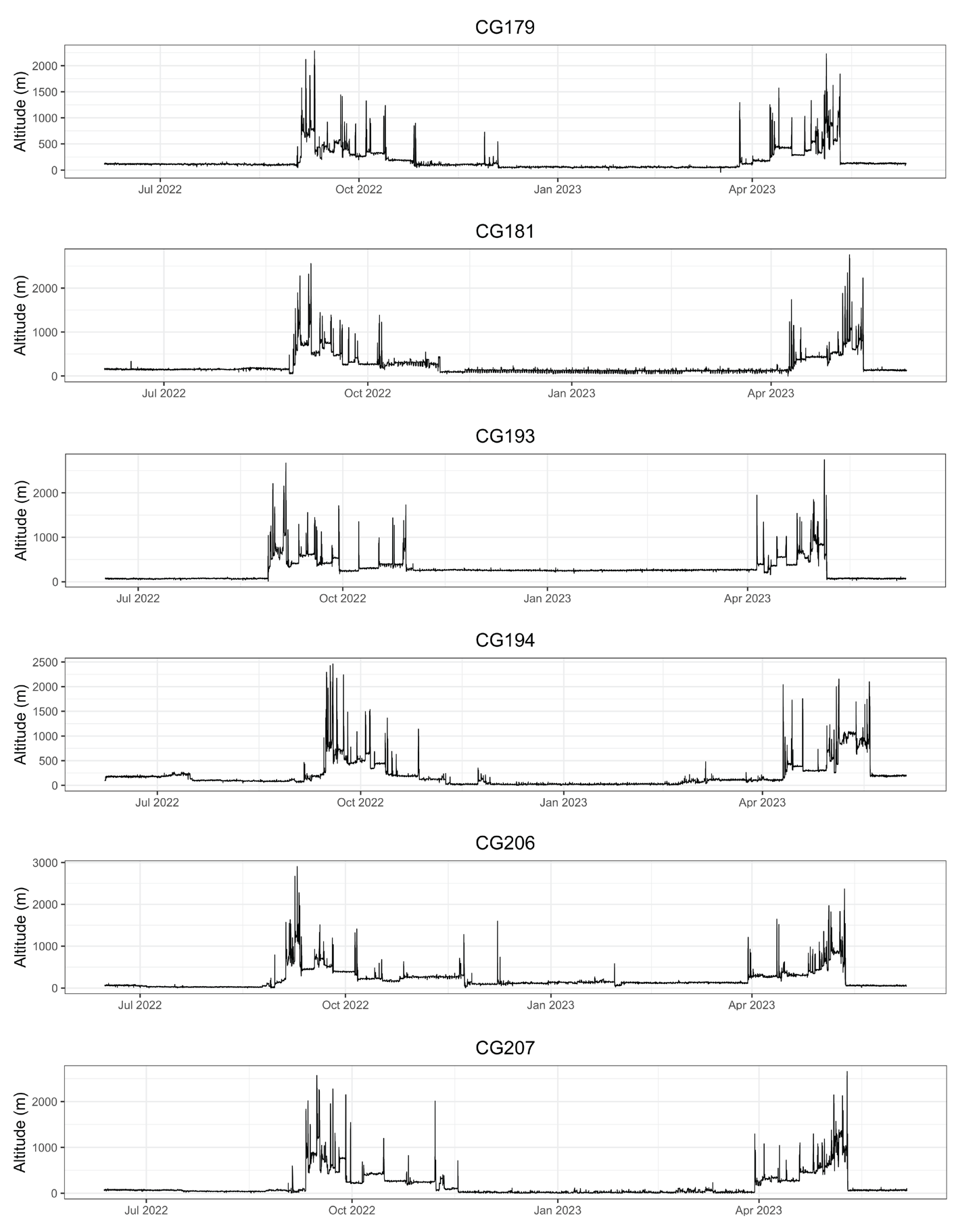


## Figure S18. Altitude over the full year for six myrtle warblers determined from atmospheric pressure data recorded by geolocators.


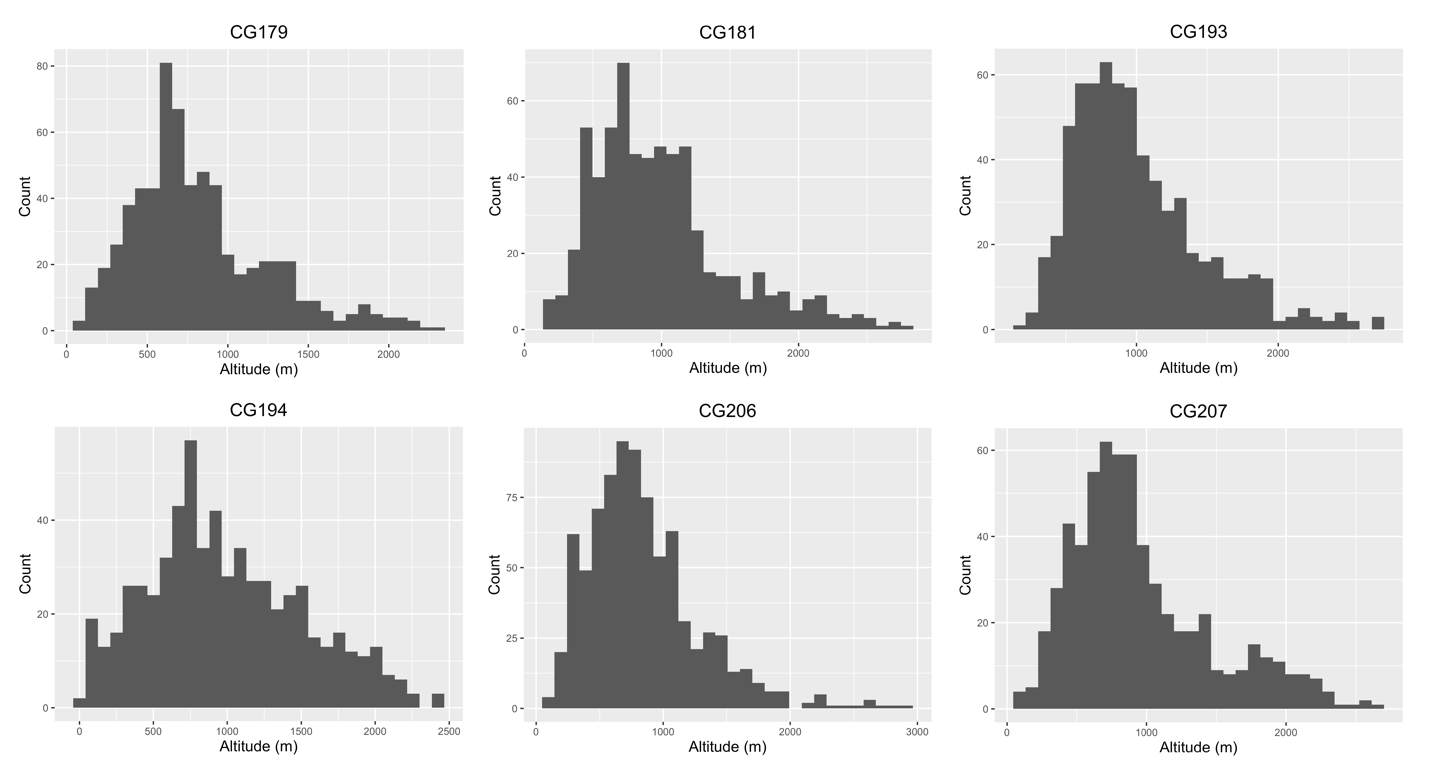


Figure S19. Histograms depicting distributions of flight altitude for six myrtle warblers determined from atmospheric pressure data collected by geolocators. Pressure readings were recorded every 20 minutes to characterize changes in altitude over the course of the flight.


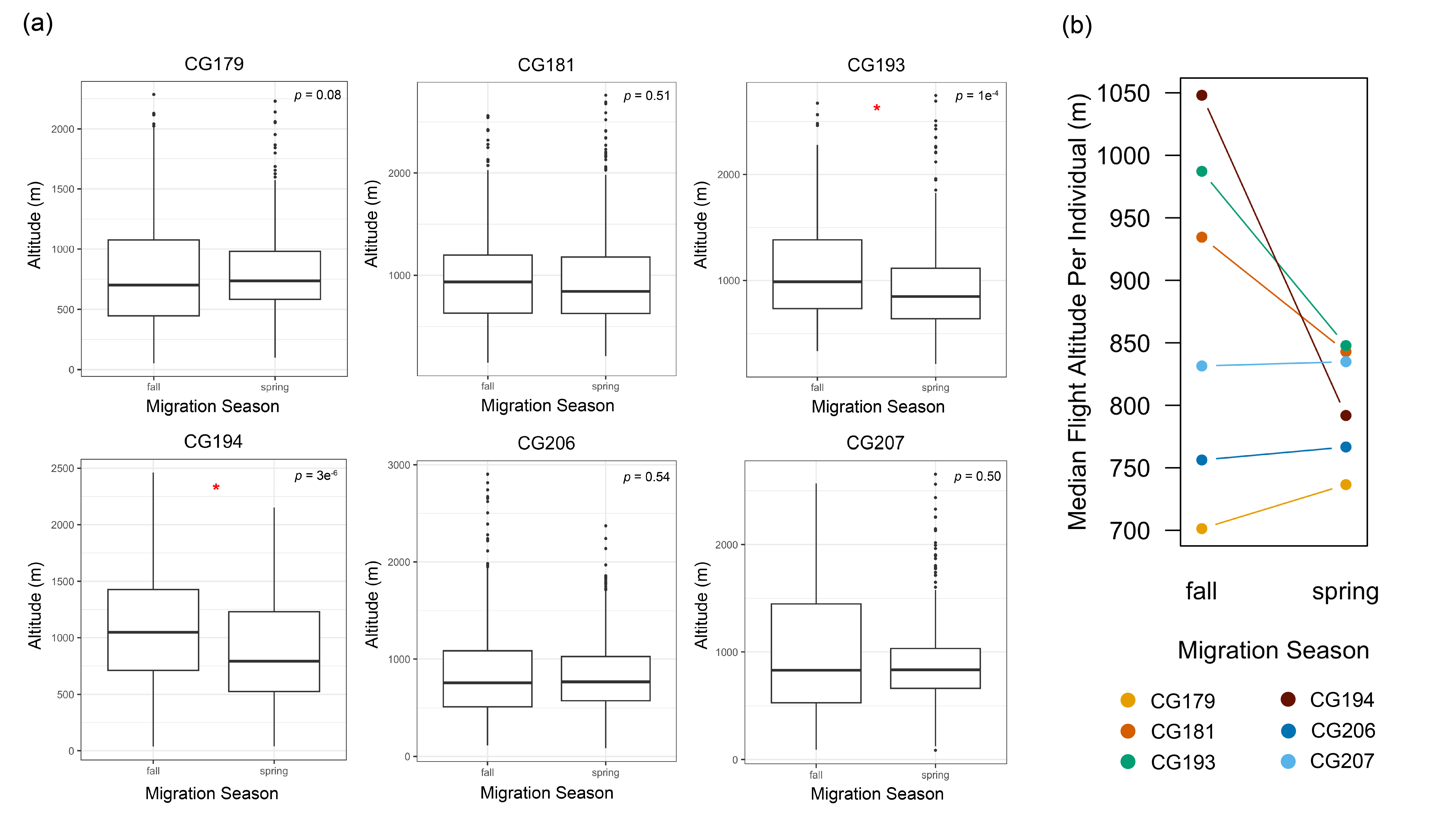


Figure S20. Flight altitude during fall and spring migration for six myrtle warblers determined from atmospheric pressure data collected by geolocators. (a) Boxplots showing distribution of flight altitudes in fall and spring migration for each bird. Pressure readings were recorded every 20 minutes to characterize changes in altitude over the course of a flight. Flight altitudes were significantly lower in spring compared to fall for two birds (indicated by red asterisks): CG193 (Wilcoxon rank sum test *W* = 62832, *p* = 0.0001) and CG194 (*W* = 58425, *p* = 3e^-6^). (b) Interaction plot showing median flight altitude during fall vs. spring migration for each bird.

**
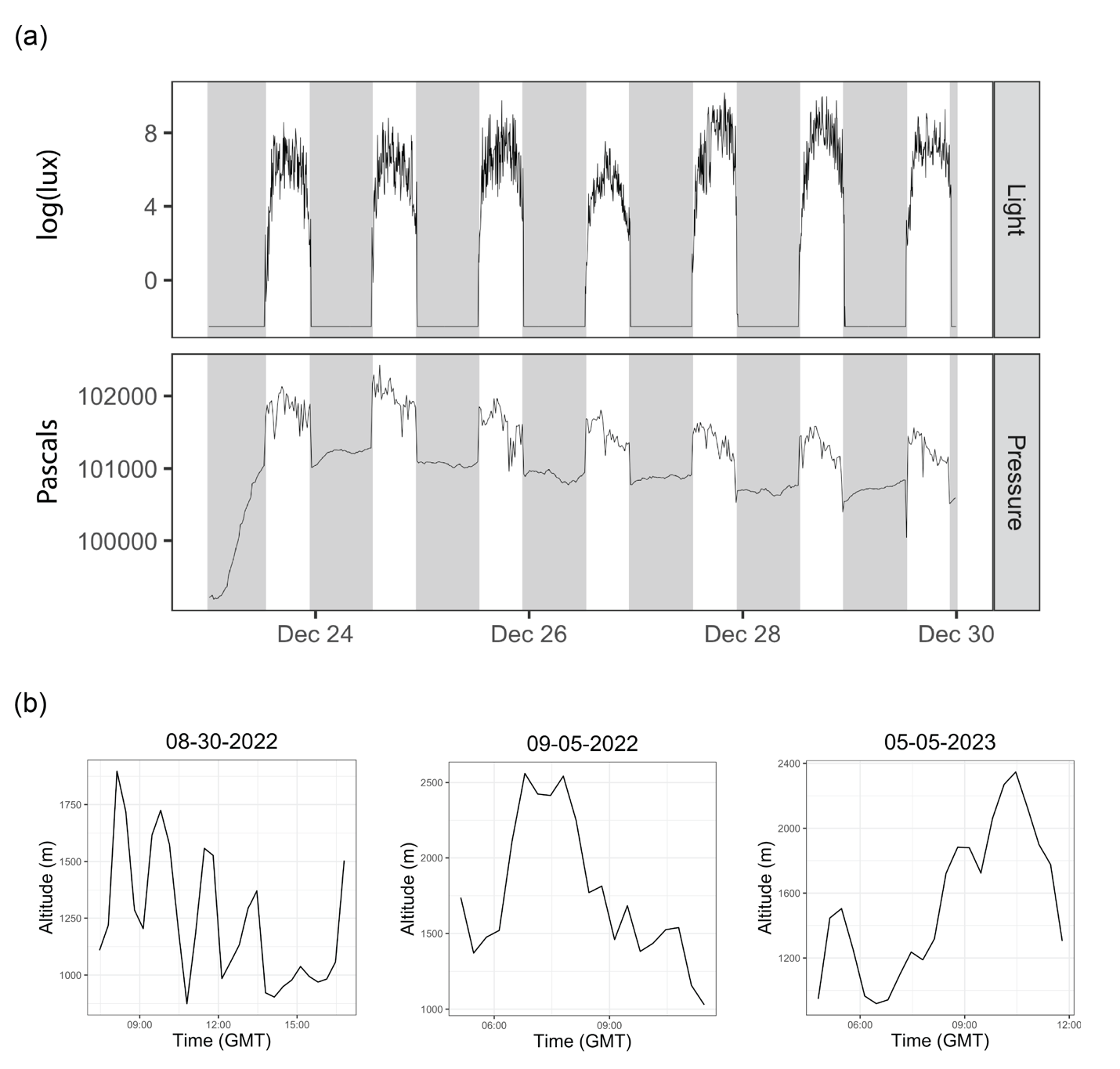
**

Figure S21. Fine scale changes in altitude observed from atmospheric pressure data collected by geolocators. (a) Over the nonbreeding period, some birds exhibited cyclical changes in pressure that corresponded to periods of daylight and darkness, likely indicative of vertical movements related to foraging and roosting. (b) During a single flight, changes in pressure showed that birds flew at various altitudes throughout the flight.


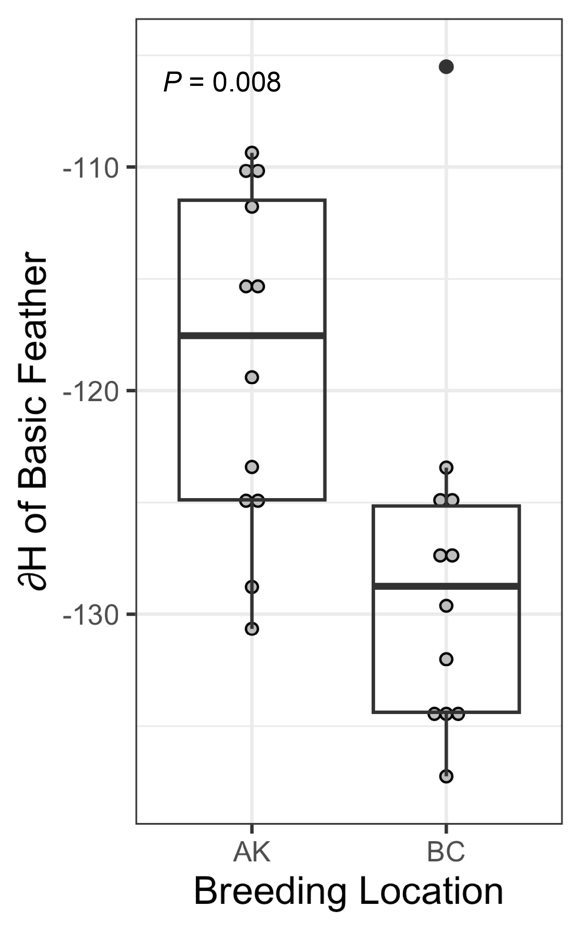


Figure S22. Comparison of stable hydrogen isotope values (∂^2^H) from basic feathers (likely grown on the previous year’s breeding ground) of myrtle warblers breeding in Anchorage, AK vs. northern British Columbia. Feathers from birds breeding in British Columbia had significantly lower ∂^2^H values than those from Alaska birds (W = 117, *p* = 0.008).

**
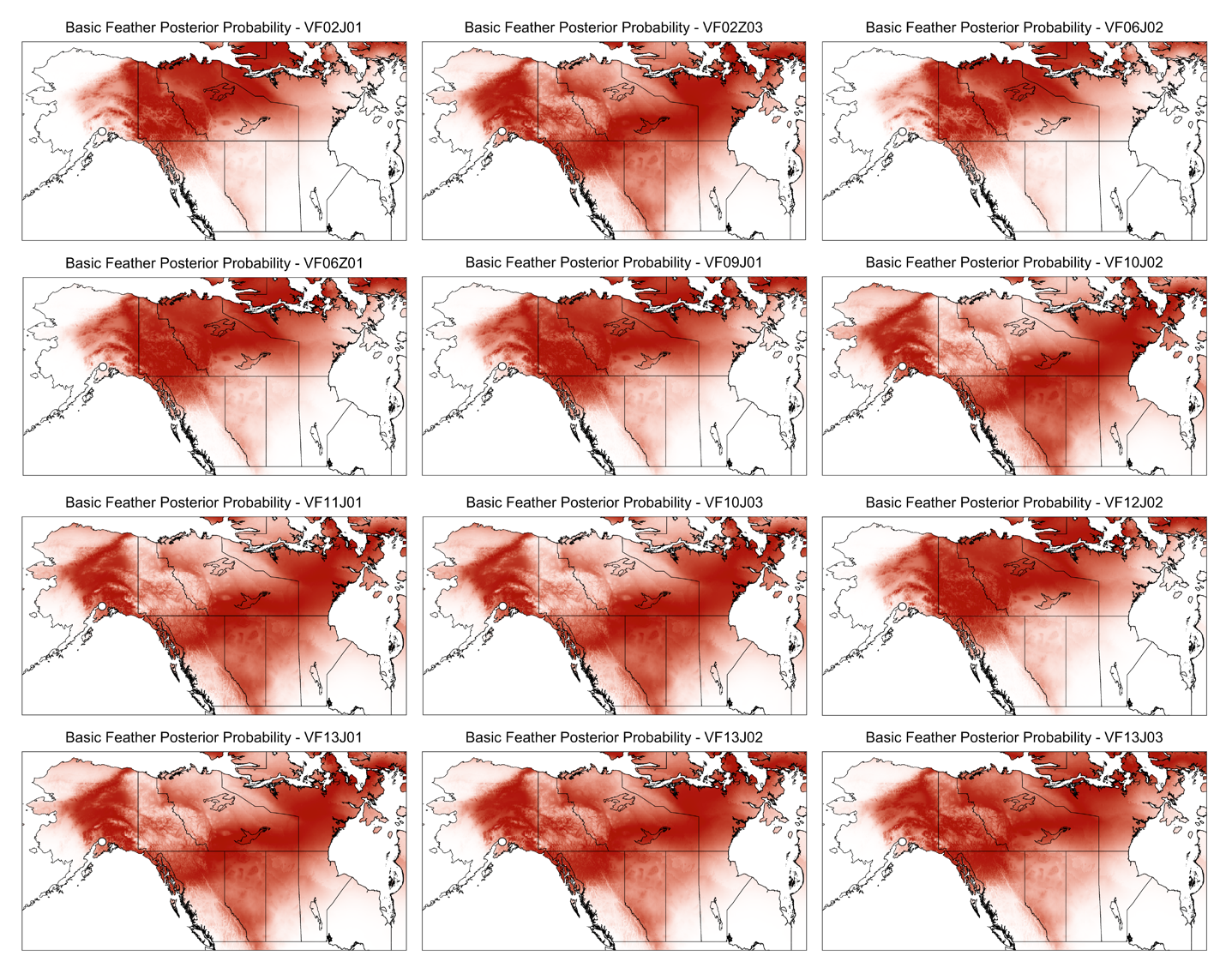
**

Figure S23. Stable hydrogen isotope posterior probability density maps for greater covert feathers grown in the pre-basic molt (likely on the previous year’s breeding ground) of myrtle warblers breeding in Alaska. The white circle marks the sampling location, and darker red color indicates greater probability of origin.

**
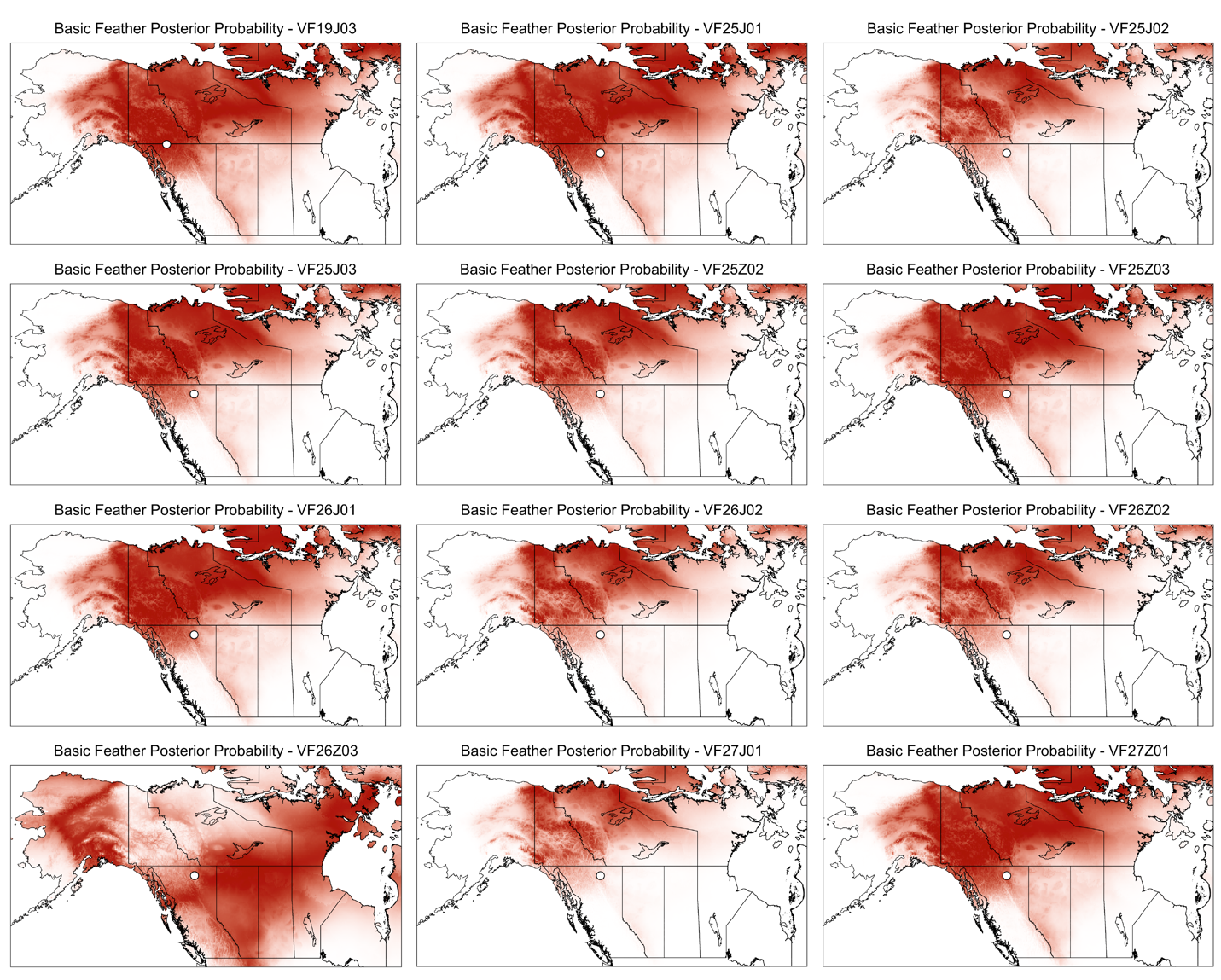
**

Figure S24. Stable hydrogen isotope posterior probability density maps for greater covert feathers grown in the pre-basic molt (likely on the previous year’s breeding ground) of myrtle warblers breeding in northern British Columbia. The white circle marks the sampling location, and darker red color indicates greater probability of origin.

**
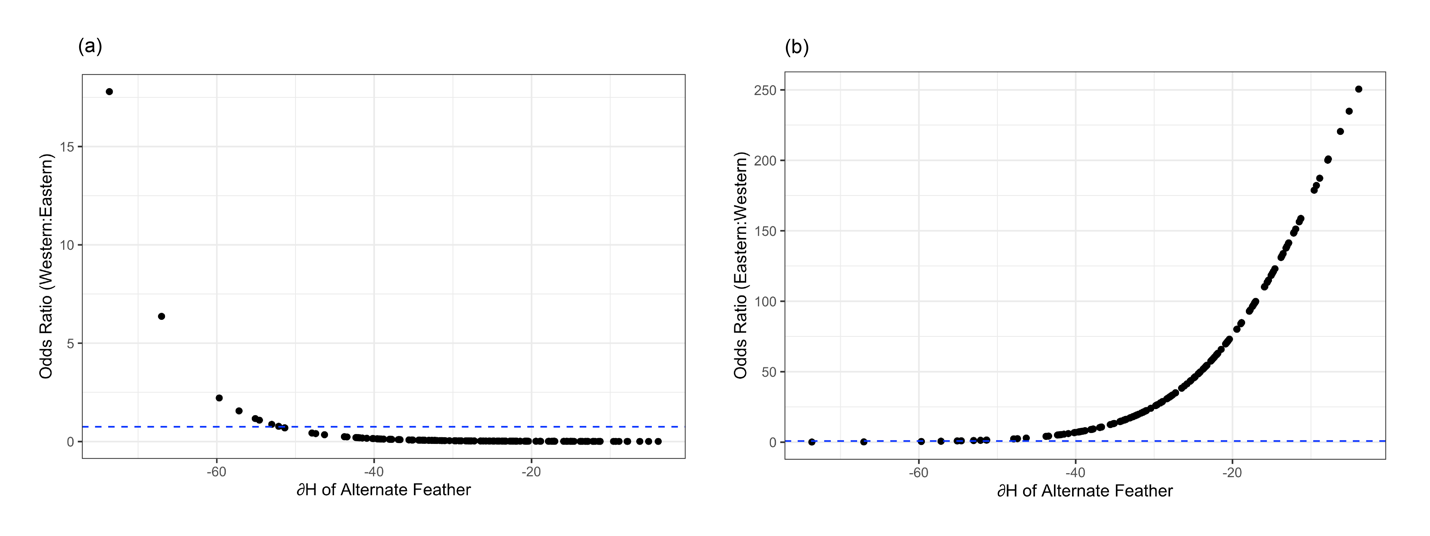
**

Figure S25. Odds ratios for wintering on the Pacific Coast (“western”) vs. Gulf Coast (“eastern”) nonbreeding areas for 167 myrtle warblers comparing posterior probabilities of origin for alternate covert feathers based on stable hydrogen isotopes. The dotted blue line indicates the ratio of the geographical areas of the two potential nonbreeding regions (0.75)—samples with an odds ratio of 0.75 are equally likely to have originated from either region. In (a) points above the blue dotted line represent feather samples with higher odds of originating from the western nonbreeding area, and in (b) points above the dotted line are samples with higher odds of originating from the eastern nonbreeding area.


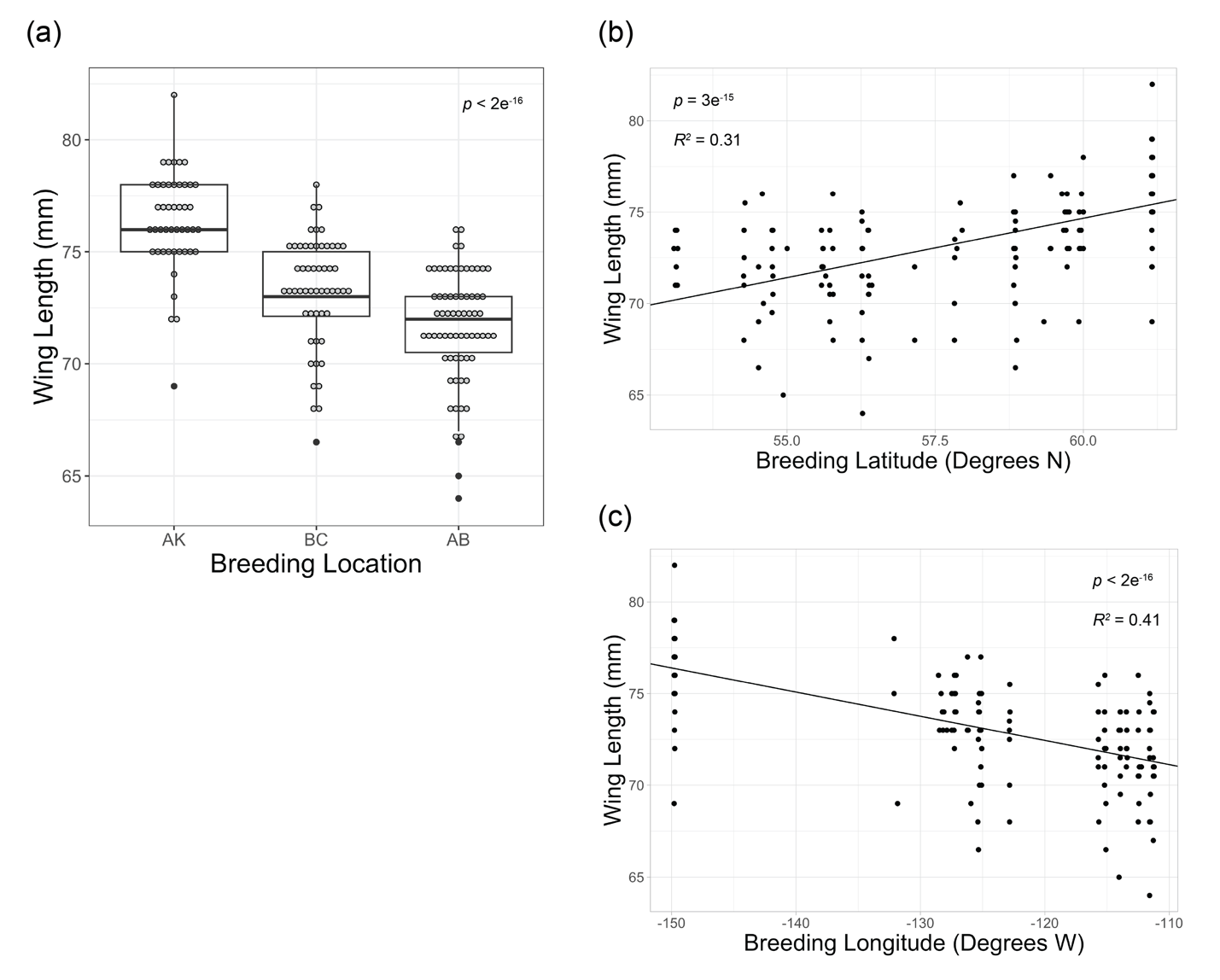


Figure S26. Relationship between wing length and breeding location for 167 myrtle warblers breeding in northwestern North America. (a) Wing length was significantly different between the three broad sampling areas: Alaska, British Columbia, and Alberta (Kruskal-Wallis χ^2^ = 73.418, *df* = 2, *p* < 2.2e^-16^; Dunn’s Test all pairwise comparisons *p* < 0.008). (b) There was a significant positive relationship between wing length and breeding latitude (*p* = 3e^-15^, *R*^2^ = 0.31). (c) There was a significant negative relationship between wing length and breeding longitude (*p* < 2e^-16^, *R*^2^ = 0.41).


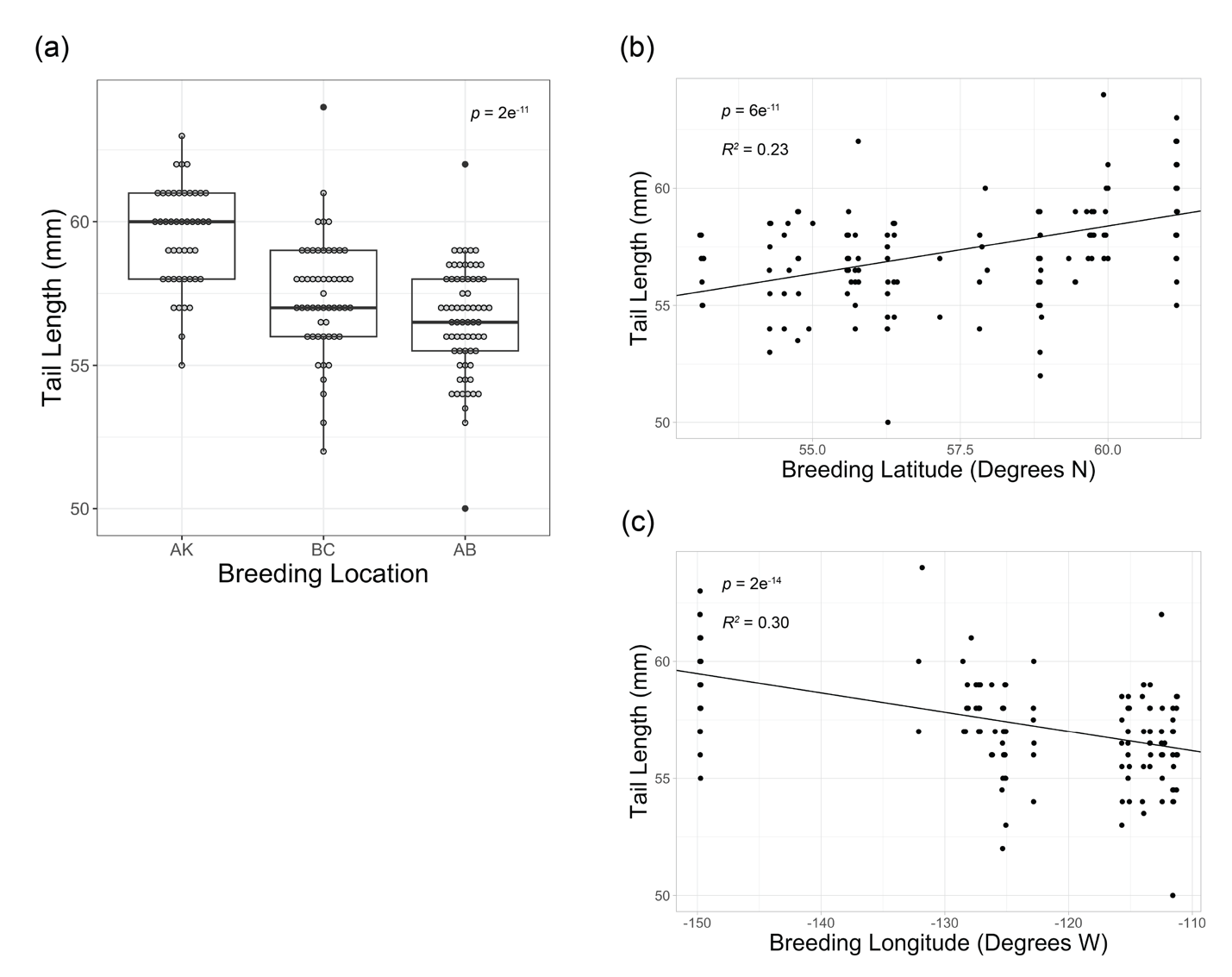


Figure S27. Relationship between tail length and breeding location for 167 myrtle warblers breeding in northwestern North America. (a) There was a significant difference in tail length among the three broad sampling areas (Kruskal-Wallis χ^2^ = 49.527, *df* = 2, *p* = 1.8e^-11^). Tail length was significantly longer in Alaska than in British Columbia (Dunn’s Test of pairwise comparisons *p* = 7e^-6^) or Alberta (*p* = 9e^-12^), but not significantly different between British Columbia and Alberta (*p* = 0.11). (b) There was a significant positive relationship between tail length and breeding latitude (*p* = 6e^-11^, *R*^2^ = 0.23). (c) There was a significant negative relationship between tail length and breeding longitude (*p* = 2e^-14^, *R*^2^ = 0.30).

**
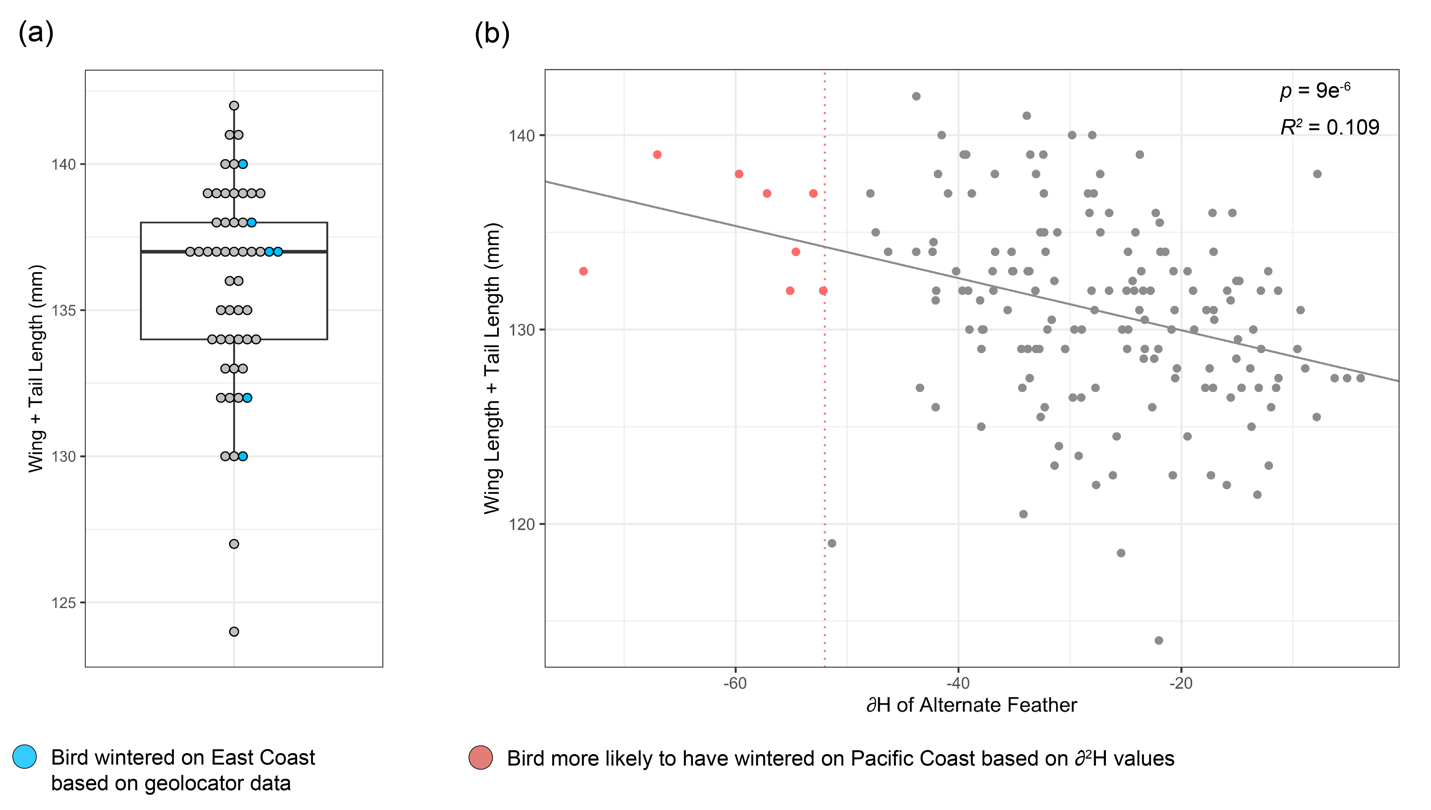
**

Figure S28. Relationships between wing and tail length and inferred nonbreeding area. (a) Distribution of sizes (wing length + tail length) of myrtle warblers banded in Anchorage, AK. Blue points indicate the birds confirmed to breed on the Gulf Coast based on geolocator data. (b) Relationship between size (wing length + tail length) and stable hydrogen isotope ratio (∂^2^H) for all 167 myrtle warblers. All birds with ∂^2^H values suggestive of Pacific Coast wintering (pink shading) had long wings and tails (i.e. “*hooveri*” type), but birds more likely to winter on the East Coast (grey) exhibited a range of sizes including many birds that historically would have been categorized as *S. c. hooveri*.

**
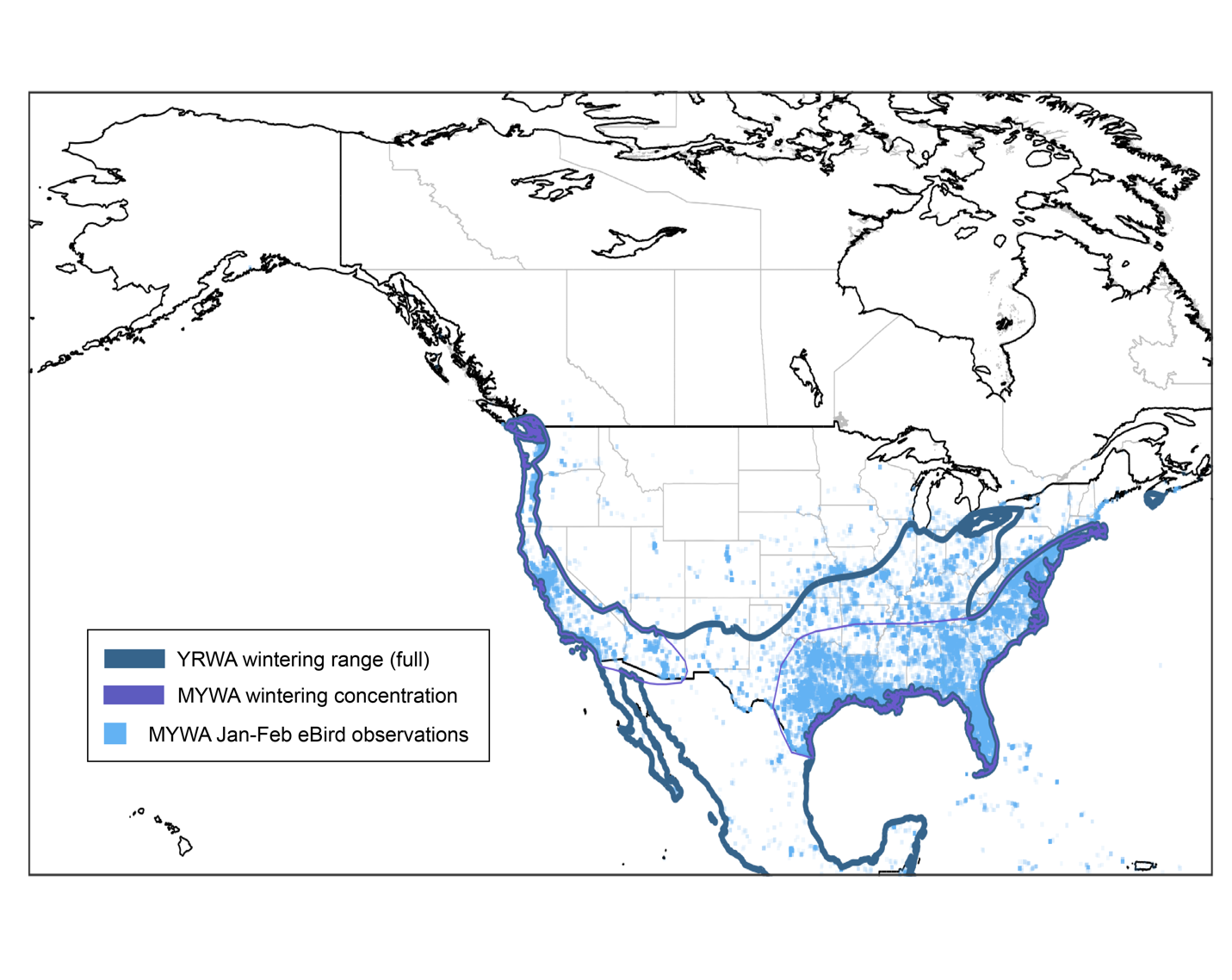
**

Figure S29. Map of myrtle warbler nonbreeding areas. The dark blue outline represents the full myrtle warbler nonbreeding range, modified from BirdLife International yellow-rumped warbler range shapefiles. The purple outlines surround the regions of greatest myrtle warbler winter abundance, based on eBird observations (blue points) and the eBird Status and Trends abundance map. These nonbreeding concentration polygons were used for assessing differences in precipitation stable hydrogen ratios (∂^2^H) between the two nonbreeding areas, and for calculating odds ratios of feather origins from the East Coast versus West Coast.

**
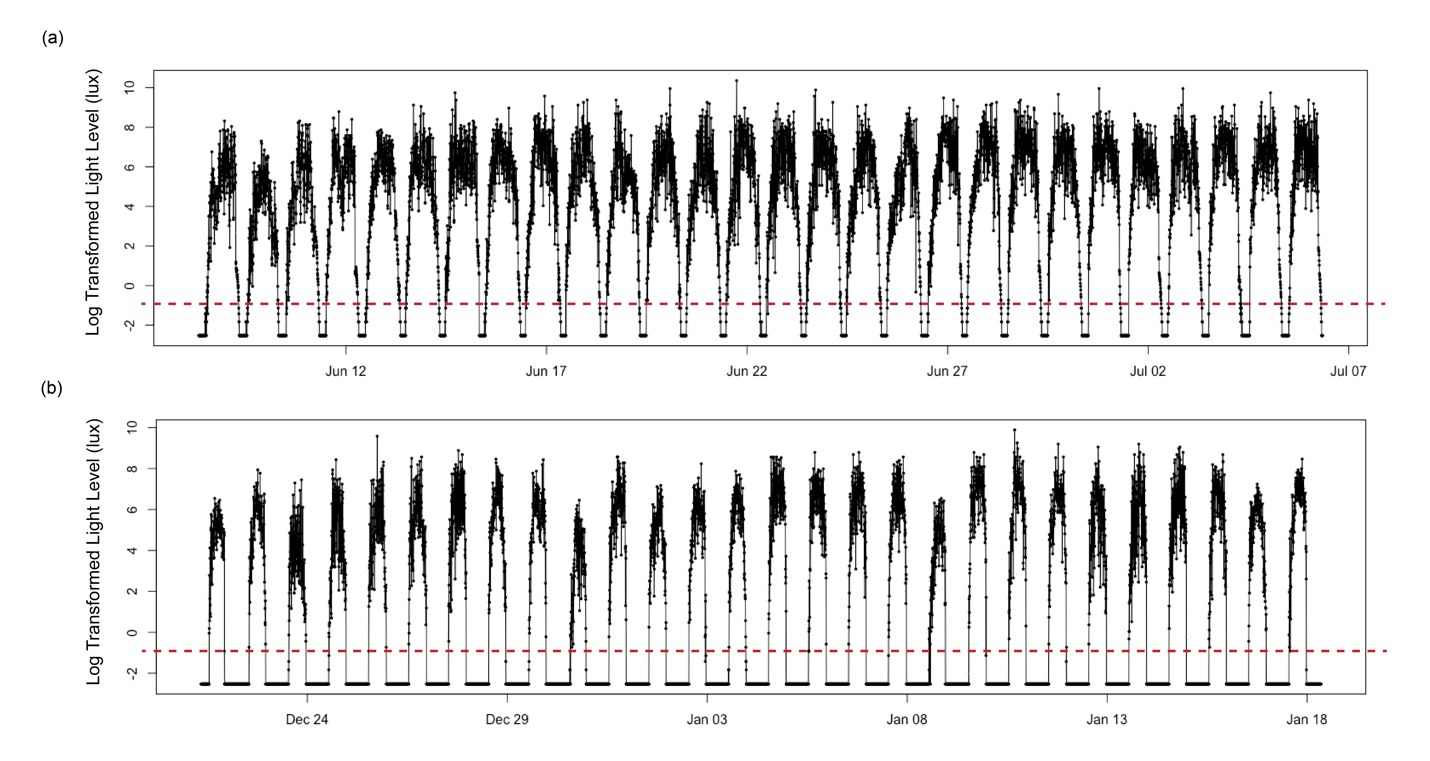
**

Figure S30. Light level data from one geolocator over two time periods: (a) summer on the breeding ground (9 June 2022—6 July 2022), and (b) winter on the non-breeding ground (22 December 2023—18 January 2023). The twilight threshold used for GeoLight analyses is shown by the dashed red line. Light data is log transformed to improve visualization.

**
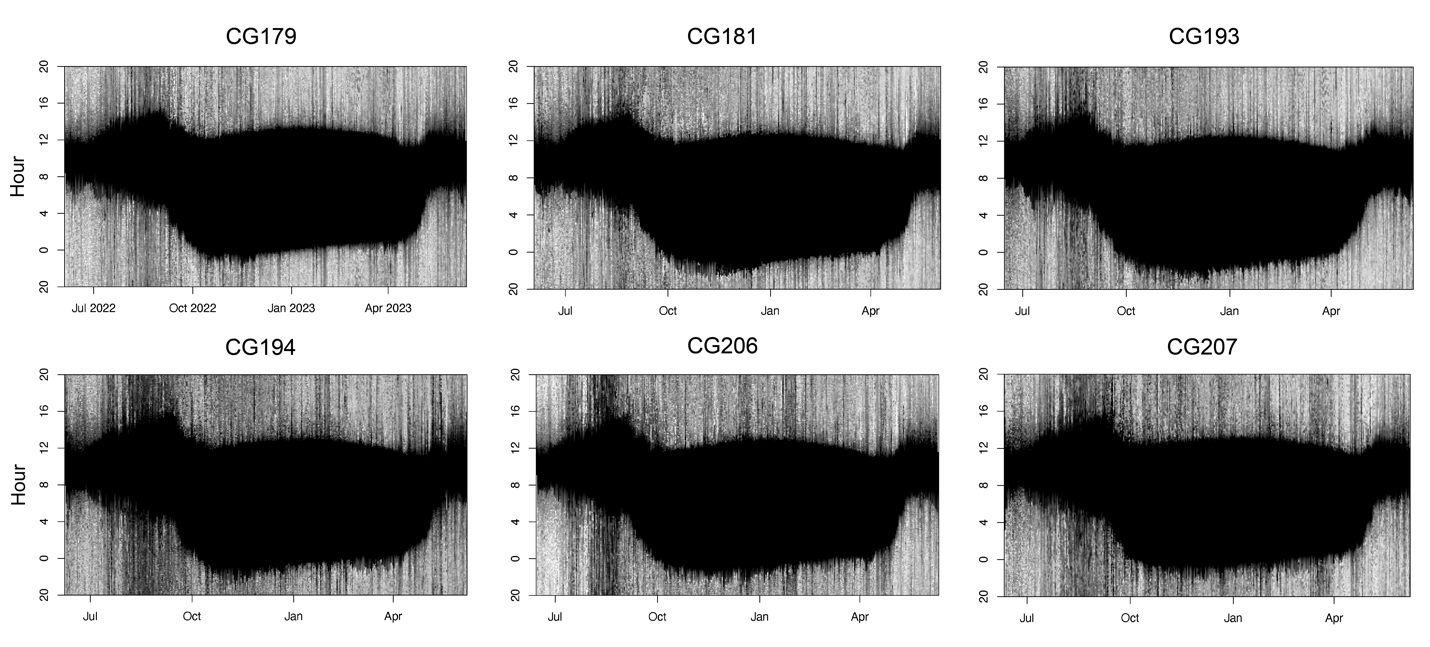
**

Figure S31. Light level measured over the full year for each of six geolocators. Each vertical line represents light levels over one day, with hours of the day represented along the y-axis. The nighttime period has been centered in each plot. Dark pixels represent complete darkness, while increasingly white pixels represent higher levels of light. Sudden shifts in the timing of darkness (night) are indicative of movement to a different location.

**
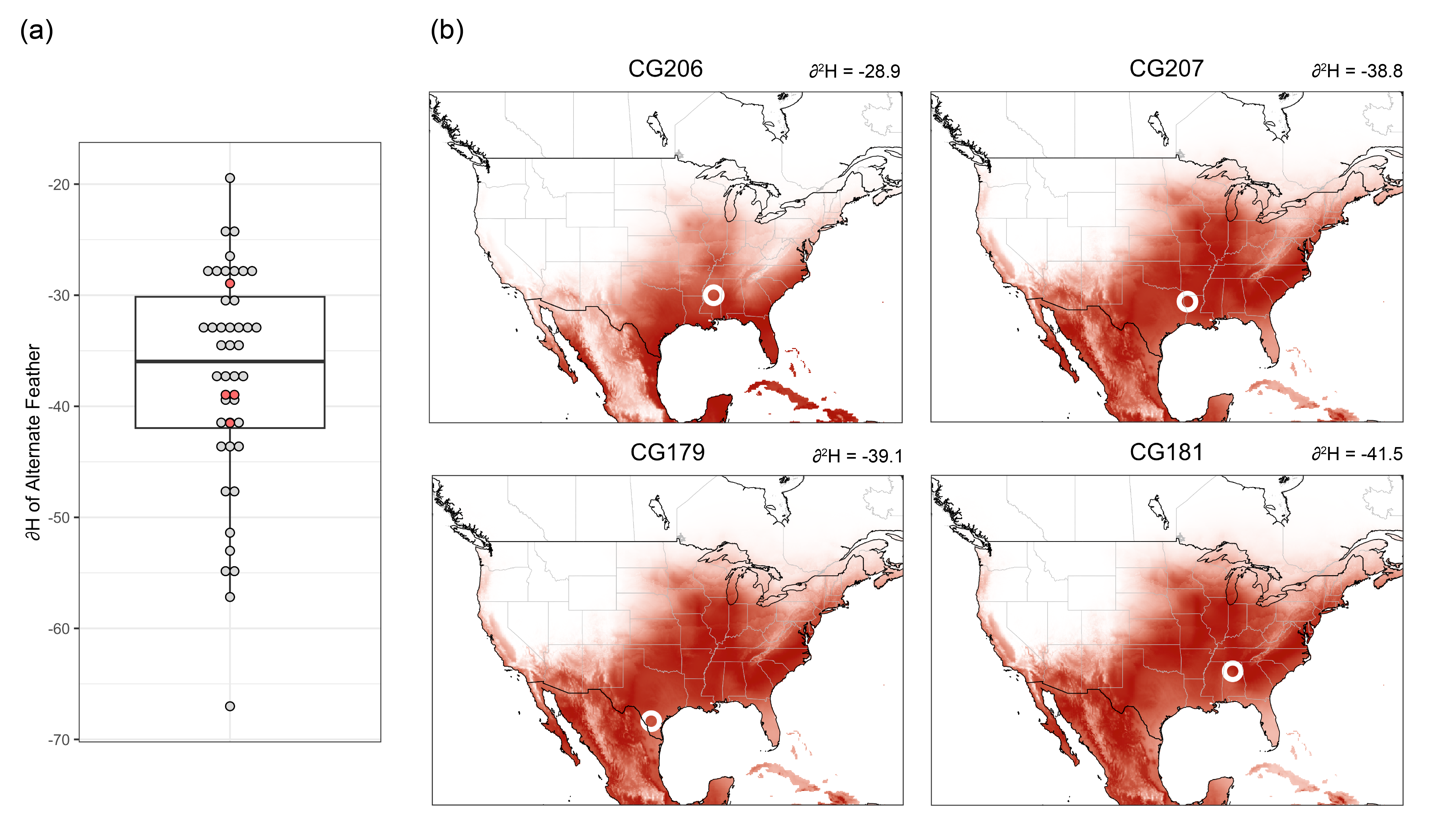
**

Figure S32. Stable hydrogen isotope ratios from feathers of myrtle warblers also tracked using geolocators. (a) Distribution of ∂^2^H for all birds sampled from Anchorage, AK with birds that also had geolocator data shaded in red. (b) Isotope likelihood surfaces for the four birds that had both isotope and geolocator data. Nonbreeding areas inferred from geolocators are marked with a white ring.

# SUPPLEMENTARY TABLES

## Table S1. Parameters used in estimation of twilight times and calibration of light-level geolocator data.

| Geolocator | Latitude of Deployment | Longitude of Deployment | Date of Deployment | Calibration Start Date | Calibration End Date | Light Threshold (lux) | Sun elevation angle (median) |
| --- | --- | --- | --- | --- | --- | --- | --- |
| CG179 | 61.154597 | -149.738660 | 3 June | 4 June | 25 June | 1.00 | -3.43 |
| CG181 | 61.158430 | -149.750052 | 2 June | 3 June | 24 June | 1.00 | -3.52 |
| CG193 | 61.165691 | -149.779153 | 14 June | 15 June | 6 July | 0.37 | -2.43 |
| CG194 | 61.162198 | -149.725350 | 7 June | 8 June | 29 June | 0.37 | -3.45 |
| CG206 | 61.157656 | -149.793144 | 13 June | 14 June | 5 July | 0.37 | -4.07 |
| CG207 | 61.159740 | -149.780571 | 11 June | 11 June | 2 July | 0.37 | -3.66 |

Table S2. Point estimates for nonbreeding areas of six myrtle warblers inferred using multi-sensor geolocators. Coordinates reported for GeoLight analysis are the average latitude and longitude of all point estimates generated using the threshold method over the longest winter stationary period. Coordinates generated using the GeoPressureR method represent the grid cell with the highest marginal probability for the trajectory model incorporating both light and pressure data. Also reported are the differences in latitude and longitude between the estimates from each method.

|  | GeoLight | | GeoPressureR | | Latitude difference between methods | Longitude difference between methods |
| --- | --- | --- | --- | --- | --- | --- |
| Geolocator | Latitude | Longitude | Latitude | Longitude |  |  |
| CG179 | 26.774 | -98.841 | 28.071 | - 98.929 | 1.30 | -0.09 |
| CG181 | 32.726 | -86.382 | 33.900 | -86.900 | 1.17 | -0.52 |
| CG193 | 35.132 | -81.241 | 35.083 | -81.917 | -0.05 | -0.68 |
| CG194 | 32.203 | -90.909 | 30.900 | -90.900 | -1.30 | 0.01 |
| CG206 | 37.632 | -89.008 | 33.100 | -89.500 | -4.53 | -0.49 |
| CG207 | 32.515 | -92.660 | 32.300 | -93.500 | -0.22 | -0.84 |

Table S3. Statistics describing migration timing for six myrtle warblers tracked using multi-sensor geolocators. Timing estimates were derived from atmospheric pressure data.

| Geolocator | CG179 | CG181 | CG193 | CG194 | CG206 | CG207 |
| --- | --- | --- | --- | --- | --- | --- |
| *Fall migration* | | | | | | |
| Breeding site departure | 2 Sep | 29 Aug | 28 Aug | 5 Sep | 30 Aug | 4 Sep |
| Number of migratory flights | 18 | 16 | 19 | 19 | 19 | 19 |
| Average flight duration (hours) | 5.3 | 6.4 | 5.6 | 5.7 | 6.3 | 5.3 |
| Maximum flight duration (hours) | 8.7 | 10.7 | 10.0 | 11.7 | 13.0 | 13.7 |
| Average stopover length (days) | 5.2 | 4.1 | 3.2 | 4.5 | 4.7 | 3.9 |
| Total stopover time (days) | 89 | 61 | 58 | 80 | 81 | 70 |
| Wintering site arrival | 4 Dec | 2 Nov | 29 Oct | 28 Nov | 23 Nov | 17 Nov |
| Fall migration duration (days) | 93 | 65 | 62 | 84 | 85 | 74 |
| *Wintering period* | | | | | | |
| Days at wintering site | 111 | 158 | 158 | 89 | 127 | 133 |
| *Spring migration* | | | | | | |
| Wintering site departure | 26 March | 9 April | 5 April | 25 Feb | 30 March | 30 March |
| Number of migratory flights | 16 | 14 | 12 | 17 | 18 | 14 |
| Average flight duration (hours) | 7.6 | 8.1 | 9.4 | 6.1 | 8.9 | 7.6 |
| Maximum flight duration (hours) | 15.0 | 12.0 | 17.7 | 13.7 | 17.0 | 14.0 |
| Average stopover length (days) | 2.8 | 2.2 | 2.4 | 5.0 | 2.2 | 2.9 |
| Total stopover time (days) | 42 | 29 | 27 | 80 | 37 | 37 |
| Breeding site arrival | 11 May | 12 May | 6 May | 19 May | 12 May | 10 May |
| Spring migration duration (days) | 47 | 33 | 31 | 83 | 44 | 42 |

Table S4. Standard information for stable hydrogen isotope analysis at the Cornell University Stable Isotope Laboratory. Reported are mean and standard deviation ∂^2^H vs. VSMOW for three standards over three different runs.

| Run | CBS | | KHS | | Keratin | |
| --- | --- | --- | --- | --- | --- | --- |
|  | mean ∂^2^H | SD ∂^2^H | mean ∂^2^H | SD ∂^2^H | mean ∂^2^H | SD ∂^2^H |
| 1 | -156.95 | 2.43 | -35.35 | 1.04 | -47.30 | 2.19 |
| 2 | -156.90 | 2.88 | -35.40 | 2.50 | -47.26 | 2.83 |
| 3 | -156.92 | 2.68 | -35.38 | 2.14 | -49.68 | 2.49 |
